# Appropriate tension sensitivity of α-catenin ensures rounding morphogenesis of epithelial spheroids

**DOI:** 10.1247/csf.22014

**Published:** 2022-06-22

**Authors:** Ryosuke Nishimura, Kagayaki Kato, Misako Saida, Yasuhiro Kamei, Masahiro Takeda, Hiromi Miyoshi, Yutaka Yamagata, Yu Amano, Shigenobu Yonemura

**Affiliations:** 1 Department of Cell Biology, Graduate School of Medical Sciences, Tokushima University, Tokushima, Tokushima, Japan; 2 Exploratory Research Center on Life and Living Systems (ExCELLS), National Institutes of Natural Sciences, Okazaki, Aichi, Japan; 3 Spectrography and Bioimaging Facility, National Institute for Basic Biology, Okazaki, Aichi, Japan; 4 Department of Basic Biology, School of Life Science, The Graduate University for Advanced Studies (SOKENDAI), Okazaki, Aichi, Japan; 5 Ultra High Precision Optics Technology Team/Advanced Manufacturing Support Team, RIKEN, Wako, Saitama, Japan; 6 Center for Advanced Photonics, RIKEN, Wako, Saitama, Japan; 7 Applied Mechanobiology Laboratory, Faculty of Systems Design, Tokyo Metropolitan University, Hachioji, Tokyo, Japan; 8 Department of Bioscience, Kwansei Gakuin University, Sanda, Hyogo, Japan; 9 Ultrastructural Research Team, RIKEN Center for Biosystems Dynamics Research, Kobe, Hyogo, Japan

**Keywords:** α-catenin, vinculin, adherens junction, morphogenesis, mechanotransduction

## Abstract

The adherens junction (AJ) is an actin filament-anchoring junction. It plays a central role in epithelial morphogenesis through cadherin-based recognition and adhesion among cells. The stability and plasticity of AJs are required for the morphogenesis. An actin-binding α-catenin is an essential component of the cadherin-catenin complex and functions as a tension transducer that changes its conformation and induces AJ development in response to tension. Despite much progress in understanding molecular mechanisms of tension sensitivity of α-catenin, its significance on epithelial morphogenesis is still unknown. Here we show that the tension sensitivity of α-catenin is essential for epithelial cells to form round spheroids through proper multicellular rearrangement. Using a novel *in vitro* suspension culture model, we found that epithelial cells form round spheroids even from rectangular-shaped cell masses with high aspect ratios without using high tension and that increased tension sensitivity of α-catenin affected this morphogenesis. Analyses of AJ formation and cellular tracking during rounding morphogenesis showed cellular rearrangement, probably through AJ remodeling. The rearrangement occurs at the cell mass level, but not single-cell level. Hypersensitive α-catenin mutant-expressing cells did not show cellular rearrangement at the cell mass level, suggesting that the appropriate tension sensitivity of α-catenin is crucial for the coordinated round morphogenesis.

## Introduction

The epithelial tissue that separates the inside and outside of the living environment contributes to a significant part of morphogenesis. It is a sheet-like structure composed of epithelial cells connected by several types of cell-cell junctions. The epithelial sheet can stretch, bend, or partly protrude to form basic structures common among species, such as grooves, hollow structures, and branching (Honda, 2017). Experiments on the separation and reassembly of sea urchin embryos or sponges indicated that cells can sort themselves depending on tissue specificity and spontaneously reassemble their tissues (Herbst, 1900; Wilson, 1907). Differences in cell-cell adhesiveness (Steinberg, 1963) or surface tension of cells (Harris, 1976) were proposed as the primary factor causing sorting in mammalian cells. The actual morphogenesis in living organisms is more complex, and many studies have been conducted using various organisms. The studies also revealed the importance of signaling (Basson, 2012), programmed cell death (Voss and Strasser, 2020), cell division (Godard and Heisenberg, 2019), cell migration (Scarpa and Mayor, 2016), and cell deformation (Paluch and Heisenberg, 2009).

Many cellular deformations that directly contribute to large deformations of epithelial tissues depend on cytoskeletal remodeling, including actin dynamics and actomyosin contraction (Quintin *et al.*, 2008; Wang, 2021). On the other hand, external forces such as shear stress (Sabine *et al.*, 2012), osmotic pressure (Narayanan *et al.*, 2020), or gravity (Porazinski *et al.*, 2015) are also implicated in tissue deformation. Meanwhile, directed cell division and cell-cell rearrangement can alter tissue morphology (Pinheiro and Bellaïche, 2018). Recently, the involvement of these factors in epithelial morphogenesis has been intensively studied using theoretical approaches. Many mathematical models have been established to predict morphological changes in epithelial tissues in response to various stimuli (Glazier and Graner, 1993; Hirashima *et al.*, 2017; Honda and Nagai, 2014; Ishihara *et al.*, 2017; Okuda *et al.*, 2015). These experimental and theoretical investigations provided a better understanding of the cell-level responses contributing to the morphogenesis of multicellular organisms.

Adherens junctions (AJs) serve as sites for transmitting tension between cells (Lecuit and Yap, 2015; Takeichi, 2014). AJs are formed near the apical region of cell boundaries, where the actin cytoskeleton is anchored by α-catenin (Yonemura, 2017). The deformation of individual cells connected by AJs causes the deformation of the entire epithelial sheet (Martin and Goldstein, 2014). In the process of epithelial morphogenesis, requiring strong tension, such as the ventral furrow formation in *Drosophila* embryos and the neural tube in vertebrates, AJs must maintain sufficient structural stability (Vasquez and Martin, 2016). Many molecules are involved in ensuring the structural stability of AJs. Cadherins enable adhesion to neighbor cells, and α-catenin binds to the cytoplasmic region through β-catenin to form the cadherin-catenin complex (Pokutta and Weis, 2007; Takeichi, 1995). α-Catenin binds to actin filaments either directly (Desai *et al.*, 2013; Ishiyama *et al.*, 2018) or by actin-binding proteins, including vinculin (le Duc *et al.*, 2010; Miyake *et al.*, 2006; Yonemura *et al.*, 2010). α-Catenin is essential in AJ formation and actin association with the complex (Hirano *et al.*, 1992; Torres *et al.*, 1997; Vasioukhin *et al.*, 2001; Watabe *et al.*, 1994; Watabe-Uchida *et al.*, 1998). In addition to α-catenin, other actin-binding proteins, such as afadin (Matsuzawa *et al.*, 2018; Sakakibara *et al.*, 2020) or ZO-1 (Itoh *et al.*, 1997) accumulate in AJs, and actin filaments are also frequently associated with AJs in tissues. Among them, vinculin binds to α-catenin in a tension-dependent manner (le Duc *et al.*, 2010; Miyake *et al.*, 2006; Yonemura *et al.*, 2010).

Under tension-free conditions, α-catenin does not bind to vinculin since the α-helix bundle containing the specific α-helix that binds to vinculin is stabilized by intramolecular interactions and cannot interact with vinculin (Hirano *et al.*, 2018; Rangarajan and Izard, 2013). When tension larger than 5 pN is applied to the molecule, the α-helix bundle becomes unstable, allowing the vinculin-binding α-helix to be exposed and bind to vinculin (Hirano *et al.*, 2018; Yao *et al.*, 2014). It has been directly confirmed by atomic force microscopy (AFM) stretching and real-time total internal reflection fluorescence (TIRF) observation that vinculin is recruited when tension is applied to the α-catenin molecule (Maki *et al.*, 2018).

What is the physiological significance of the tension sensitivity of α-catenin? Amino acid residues important for vinculin binding and tension-dependent conformational changes have been identified based on crystal structures, and tension sensitivity mutants have been generated (Hirano *et al.*, 2018; Li *et al.*, 2015; Peng *et al.*, 2012). Using some of them, several groups have investigated the collective migration of epithelial cells, showing that the constitutive binding of vinculin to α-catenin reduces the speed of collective cell migration (Matsuzawa *et al.*, 2018; Seddiki *et al.*, 2018). Collective cell migration and local rearrangement between cells are essential for convergent extension (CE) (Tada and Heisenberg, 2012). An α-catenin mutant lacking the vinculin-binding site also decreases migration speed and prevents the completion of CE in zebrafish embryos (Han *et al.*, 2016). Thus, although the contexts differ, there is accumulating evidence on the importance of proper binding and dissociation of actin-binding proteins to α-catenin in the planar movement in cells in the same direction. However, the significance of this property of α-catenin in three-dimensional (3D) morphogenesis is almost unknown.

In this study, we used a newly developed morphogenetic model system to investigate the significance of the tension sensitivity of α-catenin in the 3D morphogenesis of epithelial cells. We performed analyses at the molecular complex, cellular, and multicellular levels. They suggested that α-catenin’s ability to dissociate actin-binding proteins in a tension-dependent manner is essential for normal AJ maturation and the spheroid rounding morphogenesis.

## Materials and Methods

### Cell culture

DLD-1 cells from ATCC, its subclone R2/7 cells (Watabe-Uchida *et al.*, 1998) (provided by F. van Roy, Ghent University, Belgium), and R2/7 cell lines expressing various α-catenin mutants were cultured in Dulbecco’s Modified Eagle Medium (DMEM; Wako) supplemented with 10% fetal bovine serum (FBS).

### Spheroid formation

For the spheroid formation in round-bottomed wells, non-adherent round-bottomed 96-well plates (EZ-BindShut SP, Iwaki) were used. Cells were seeded in each well at 100 cells/well, and cultured for 48 h. For the spheroid formation in V-bottomed rectangular microwells, polydimethylsiloxane (PDMS; Sylgard 184, Dow Corning) or CYTOP (CTX-809SP2, Asahi Glass) was shaped by a metal mold according to manufacturers’ instructions and attached to a 35 mm glass-bottomed dish (Matsunami Glass), then treated with 10% Poloxamer 188 (Pluronic F68, GE Healthcare) overnight to prevent cell attachment (Wang *et al.*, 2014; Wu and Hjort, 2009). The dish was rinsed thrice using PBS and filled with culture medium or imaging medium (FluoBrite DMEM, Thermo Fisher) supplemented with 10% FBS. A glass cloning cylinder (inner diameter 7 mm; Iwaki) was placed on the substrate to limit the area of cell seeding (1×10^5^ cells for the cell tracking and 2×10^5^ cells for all other experiments). Cells were cultured in the microwells for 48 h. For spheroid formation on an ECM gel, 10 μL Matrigel (Corning) was placed on round coverslips (diameter 14 mm, Matsunami Glass) and gelled by incubating for 10 min at 37°C. Cell suspension (1×10^5^ cells) was added on the gel, and cells were cultured for 9–24 h.

### Spheroid fusion

For spheroid fusion assays, R2/7 cell lines expressing WT or mutant α-catenin were cultured on V-bottomed square microwells (width 250 μm×height 250 μm×depth 160 μm) for 24 h to obtain relatively small spheroids. Two spheroids were picked up using a micropipette and transferred together into one well of non-adherent V-bottomed 96-well plates (EZ-BindShut SP, Iwaki). Then, the two spheroids were allowed to contact each other and were cultured for 9 h.

### Preparation of metal molds

The mold for making PDMS microwells was manufactured by ultraprecision cutting using a single crystal diamond tool with an ultrahigh precision machine (NPIC-M200, Nagase Integrex Co., Ltd.). The material of the mold was nickel-phosphate electroless plating (thickness of 300 μm) on a stainless-steel base with diameter of 10 mm and height of 6 mm. The diamond cutting tool had rectangular edge with flat end of 50 μm and nose angle of 53 degrees to form the shape of the well wall and bottom. The final depth of cut was 0.4 μm, and surface roughness of 4 nm in average was obtained. After the mold was formed by cutting, a fluorocarbon coating was deposited by a reactive ion etching machine so that PDMS can be easily removed from the mold.

### Antibodies and reagents

The following primary antibodies were used: anti-α-catenin rabbit polyclonal antibody (C2018) and anti-vinculin mouse monoclonal antibody (VIN-11-5; Sigma-Aldrich); anti-β-catenin mouse monoclonal antibody (BD Bioscience); anti-E-cadherin rat monoclonal antibody (ECCD-2; a gift from M. Takeichi, RIKEN, Japan); anti-DDDDK tag rabbit polyclonal antibody, which recognizes the Flag tag (MBL); anti-ZO-1 mouse monoclonal antibody (T8-754; a gift from Sa. Tsukita, Osaka University, Japan); anti-GAPDH rabbit monoclonal antibody (14C10; Cell Signaling Technology). Horseradish peroxidase (HRP)-conjugated goat anti-rabbit IgG antibody (Kirkegaard & Perry Laboratories) was used as a secondary antibody for immunoblotting. MagicMark XP Western Protein Standard (Thermo Fisher) was used as a protein marker. Alexa Fluor 488- conjugated donkey anti-mouse IgG, Alexa Fluor 488- conjugated donkey anti-rat IgG, Alexa Fluor 555- conjugated donkey anti-mouse IgG, Alexa Fluor 555- conjugated donkey anti-rabbit IgG, Alexa Fluor 647- conjugated donkey anti-mouse IgG, or Alexa Fluor 647- conjugated donkey anti-rabbit IgG antibodies (Thermo Fisher) were used as secondary antibodies for immunofluorescent staining. Alexa Fluor 350-, 488- or 647-conjugated phalloidin (Thermo Fisher) was used for staining actin filaments. 4',6-diamidino-2-phenylindol (DAPI; Dojindo Laboratories) was used for nuclei staining.

(–)-Blebbistatin (Wako) was prepared as a 50 mM stock in DMSO and used at 50 μM. Cytochalasin D (Sigma) was prepared as a 2 mM stock in DMSO and used at 2 μM. Jasplakinolide (Calbiochem) was prepared as a 1 mM stock in DMSO and used at 10 μM. Dynasore (Santa Cruz) was prepared as an 80 mM stock in DMSO and used at 80 μM. MiTMAB (Abcam) was prepared as a 100 mM stock in water and used at 30 μM.

### Immunoblotting

Cells (2.5×10^5^ cells) were cultured for 1 day, washed twice with ice-cold PBS, and lysed with lysis buffer [50 mM Tris-HCl (pH 7.5), 150 mM NaCl, 0.1% SDS, 1% TritonX-100, 1% Sodium deoxycholate, protease inhibitor cocktail (Nacalai)]. Proteins were resolved conventionally by SDS-PAGE and western blotting. Densitometric quantification of the immunoblotted bands was performed using the Gel Analyzer module in ImageJ.

### Immunostaining

Fixed samples were processed conventionally for immunofluorescence microscopy as previously described (Miyake *et al.*, 2006; Watanabe *et al.*, 2007). For staining of spheroids, spheroids formed in V-bottomed microwells were collected in microtubes. The duration of each operation was: fixation (30 min), permeabilization (10 min), washing (10 min; once after permeabilization, thrice after antibody incubations), primary and secondary antibody incubation (overnight at 4°C and 2 h at room temperature, respectively).

### Imaging

Images were taken using the following microscopes. For phase-contrast live-imaging, a Leica DMIRE2 inverted microscope equipped with a CCD camera (Sensicam, PCO) controlled by the software package MetaMorph version 7.7.7.0 (Molecular Devices), a stage-top CO_2_ incubator (Tokai Hit), and an N Plan 2.5×/0.07NA Ph1 lens was used. For imaging of fixed samples, 1) a Nikon A1R inverted confocal microscope controlled by Nikon NIS-Elements software (Nikon), equipped with a Plan Apo VC 60×/1.40NA or a Plan Apo Lambda 40×/0.95NA lens, or 2) an Olympus BX51 microscope equipped with a CCD camera (ORCA ER, Hamamatsu Photonics) controlled by the software package IPLab Spectrum version 3.5.4 (Scanalytics), equipped with a Plan Apo N 60x/1.42NA lens was used. For confocal live-imaging, 1) the Nikon A1R microscope equipped with a stage-top CO_2_ incubator (Tokai Hit) and a Plan Apo VC 20×/0.75NA lens, or 2) an Olympus IX71 inverted microscope equipped with a spinning disk confocal unit (CSU-X1, Yokogawa) and CMOS camera (Orca Flash 4.0, Hamamatsu Photonics) controlled by software package MetaMorph version 7.10.2.240 (Molecular Devices), a stage-top CO_2_ incubator (Tokai Hit), and a UPlan S Apo 20×/0.75NA lens was used.

### Plasmids and transfection

A vector expressing full-length mouse αE-catenin with a Flag tag at its C terminus (pCA-αE-catenin–Flag) (Abe *et al.*, 2004) was a gift from M. Takeichi (RIKEN BDR, Japan). pCA-αE-catenin-Flag (L344P) was constructed using site-specific mutagenesis to introduce a mutation resulting in the single amino acid substitution from L to P (Peng *et al.*, 2012) into pCA-αE-catenin-Flag. Stable clones expressing mutant α-catenin [1–906(L344P)] were established as previously described (Yonemura *et al.*, 2010).

The expression vector of histone H2B tagged with EGFP at the C-terminus was generated using the EGFP-N1 vector (Clontech). The amplified H2B cDNA was cloned into the HindIII/BamHI site of EGFP-N1. R2/7 cells stably expressing WT (1–906) or mutant [1–906 (L378P or L344P)] α-catenin were transfected with this vector. EGFP-expressing cells were sorted using a cell sorter (SH800, Sony), sparsely replated, and then single clones were isolated.

### Image analyses

Images were processed and analyzed using ImageJ software (National Institutes of Health) or CellProfiler software (Carpenter *et al.*, 2006). To measure spheroid morphology, the outlines of spheroids were traced by freehand drawing, and the circularity (“form factor” in CellProfiler) was calculated using the formula:

$$circularity=4\pi \frac{area}{perimeter^{2}}$$

To measure punctum adherens (PA) length, confocal Z-stack images of α-catenin and F-actin were projected to single images by maximum projection. Spot-like α-catenin-positive objects attached to F-actin bundles were considered PAs. The max Feret diameter of PA was manually measured.

To measure TJ length, confocal Z-stack images of nuclei and ZO-1 were projected as well. The EnhanceOrSuppressFeatures module of CellProfiler enhanced linear structures in the ZO-1 channel, and automated segmentation was performed to define the TJ-formed region. The TJ region was then skeletonized to single-pixel width, and the number of pixels representing the total TJ length was counted. Cell number was counted using automated nuclei segmentation, and TJ length was normalized by dividing using the cell number or field area.

To analyze cell movements during the spheroid formation, 3D DoG (Difference of Gaussian, combined Gaussian radii in sigma of 1.9/3.8 pixels for X/Y dimensions, and 0.7/1.4 pixels for Z dimension) was performed to emphasize nucleus shape and identified distinct nuclei by finding local maxima. These nuclei positions were stored in a SQL database (SQLite3), and their displacements throughout the time series were joined using the nearest neighbor method.

To analyze the morphology of spheroid doublets during fusion, doublet length and contact length measurements were recorded manually by drawing a single line along the long axis of a pair of spheroids or the fused region (the border of two spheroids), respectively.

### Statistical analyses

Data are expressed as mean±95% confidence interval (CI) of at least three independent experiments. All statistical analyses were performed in R software. *P*-values were calculated using Student’s *t*-test for comparison of two groups, and One-way ANOVA followed by Dunnett’s or Tukey’s multiple comparison tests for more than three groups, respectively. In all cases, *P*<0.05 was considered statistically significant.

## Results

### Importance of tension sensitivity of α-catenin on 3D morphogenesis of epithelial cells

**Fig. 1:**
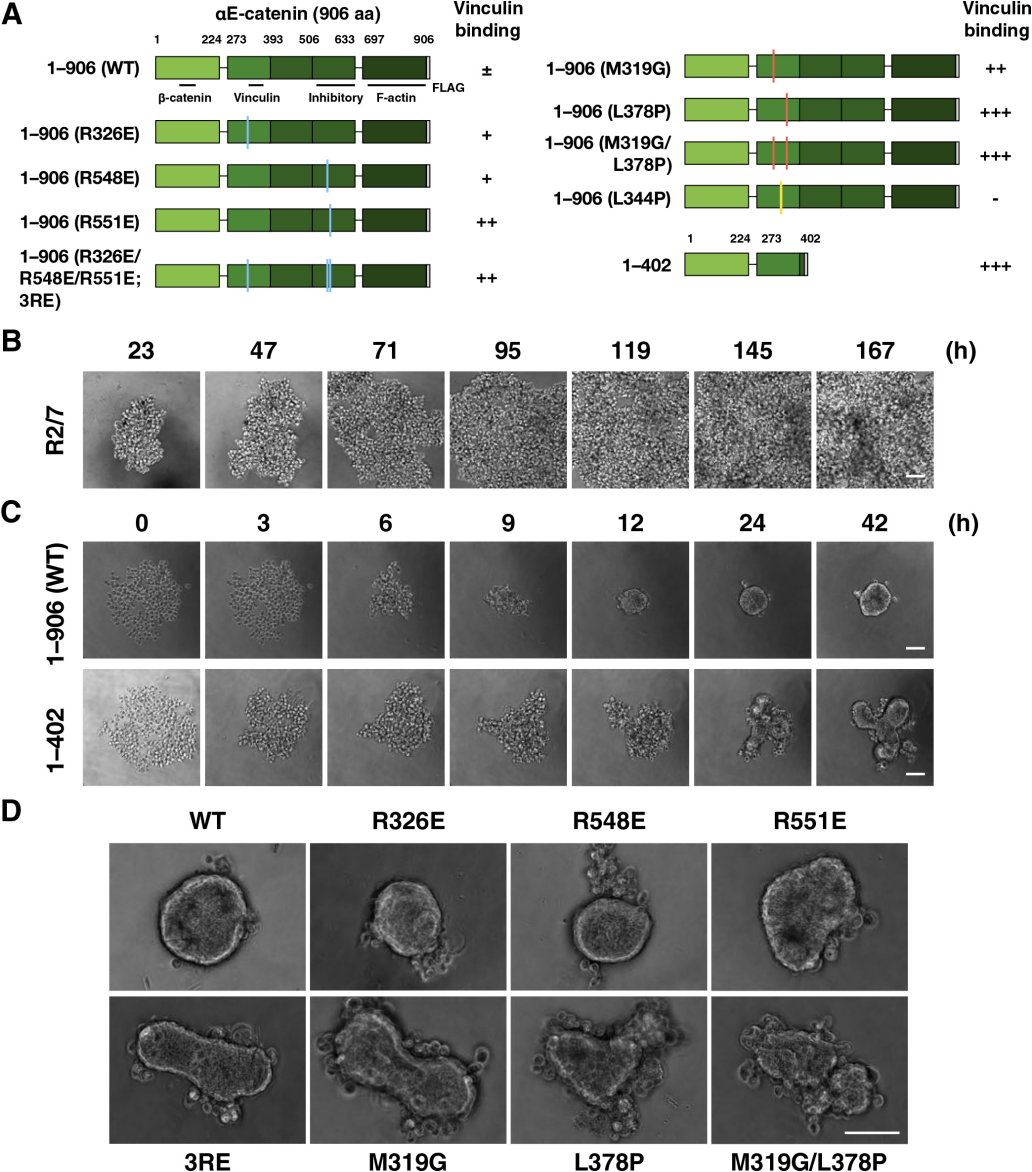
Wild-type α-catenin-expressing cells form round-shaped spheroids in round-bottomed wells, which is not supported by tension sensitivity mutants. (A) Schematic drawing of α-catenin and its mutants. The residue numbering for mouse αE-catenin is given. 1–906; full length, 1–402; C-terminus deletion. WT; wild-type, 3RE; R326E/R548E/R551E. Colored lines on the domain illustration indicate the positions of point mutations. Functional domains and strength of vinculin binding affinity are indicated. –; no binding, ±; moderate, +; strong, ++; very strong, +++; extremely strong (Hirano *et al.*, 2018). (B) Phase-contrast images of R2/7 cells cultured on round-bottomed wells for 7 days. (C) Still images by time-lapse microscopy. R2/7 cells expressing WT (1–906; top) or mutant (1–402; bottom) α-catenin were seeded on round-bottomed wells, respectively, and live-imaged for 42 h. (D) Spheroids of various mutants after 48 h culture. Scale bar, 100 μm.

To clarify the significance of the tension sensitivity of α-catenin in 3D epithelial morphogenesis, α-catenin mutants with artificially manipulated tension sensitivity for vinculin binding in the middle of the molecule (Hirano *et al.*, 2018; Peng *et al.*, 2012; Yonemura *et al.*, 2010) were expressed in R2/7 cells, an α-catenin-deficient subclone of DLD-1 cells derived from human colorectal cancer (van Hengel *et al.*, 1997; Watabe-Uchida *et al.*, 1998) (Fig. 1A). We confirmed that all mutant α-catenin molecules are colocalized with E-cadherin and β-catenin together with F-actin at the apical junction regions (Fig. S1), allowing tight junction (TJ) formation (Hirano *et al.*, 2018; Yonemura *et al.*, 2010), and show comparable levels of WT or mutant α-catenin expression (Fig. S2). These data indicate that the essential α-catenin function remains in all mutants although precise adhesion activities may differ. Under conventional two-dimensional (2D) culture conditions, only slight differences were observed in epithelial sheet formation. Therefore, a 3D culture model was used in this study to eliminate the effect of the stiff substrate on the sheets, and to see morphogenesis that reflects the nature of the AJ more strongly.

First, we tested if the tension sensitivity of α-catenin is involved in 3D epithelial morphogenesis (Fig. 1A). Cells were seeded into non-adherent round-bottomed wells, and the morphology of the cell mass was recorded over time. Parental R2/7 cells did not adhere each other, maintaining the original round shape of each cell for 7 days (Fig. 1B). R2/7 Cells expressing WT α-catenin formed a single aggregate with a smooth surface, whereas cells expressing the C-terminal deletion mutant (1–402) formed a single aggregate but with a distorted shape (Fig. 1C and Movie 1). Point mutants with different levels of increased tension sensitivity (R326E, R548E, R551E, R326/548/551E; 3RE, M319G, L378P, and M319G/L378P) (Hirano *et al.*, 2018) expressing cells also formed distorted spheroids according to the degree of enhanced tension sensitivity (Fig. 1D and Movie 2). These results indicate the importance of α-catenin tension sensitivity in spheroid morphogenesis into a round shape.

### Construction of an experimental epithelial morphogenesis model and quantitative analysis

**Fig. 2:**
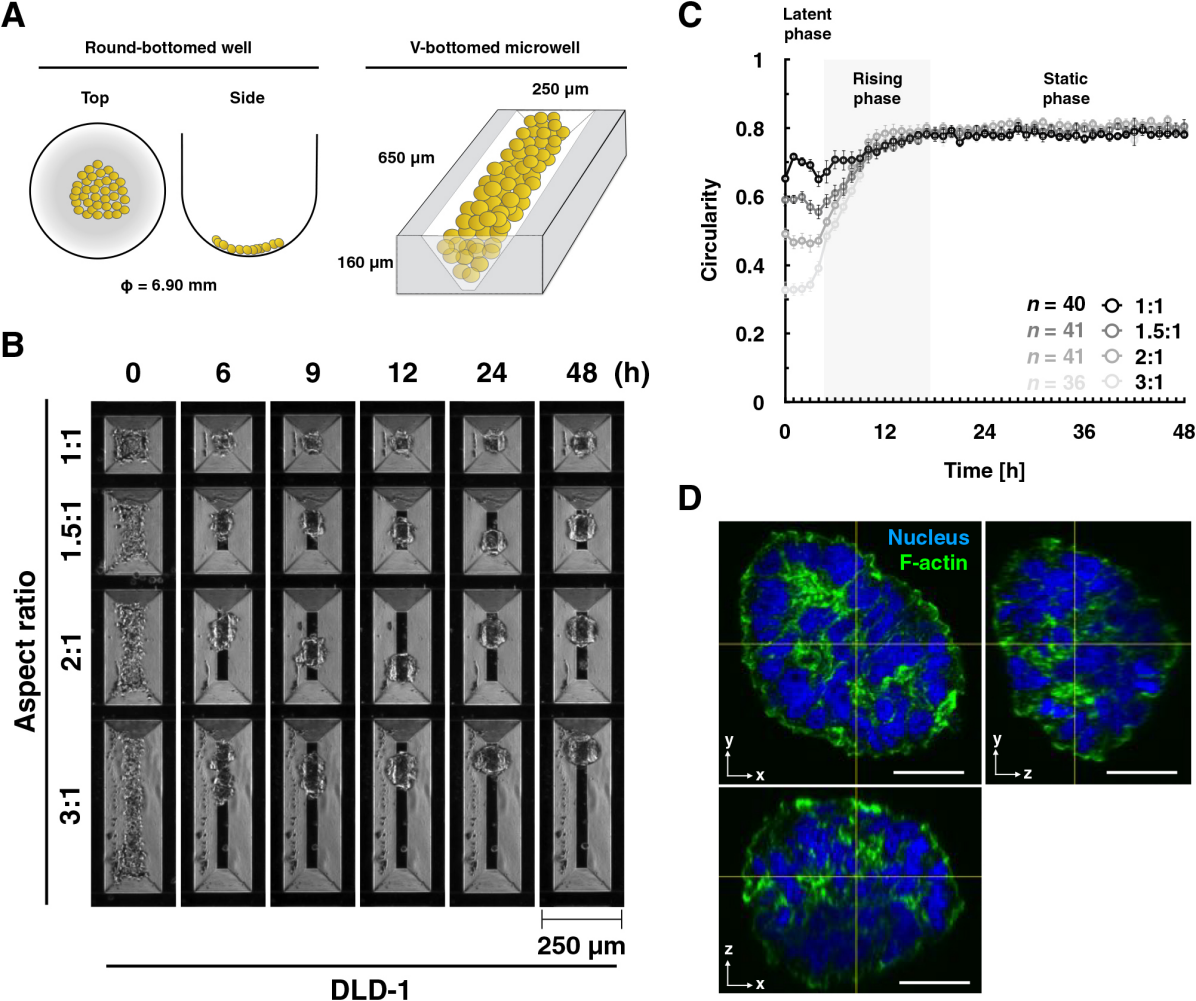
Initial aspect ratios of cell masses and final spheroid circularity. (A) Schematic drawings of a round-bottomed well and a V-bottomed microwell. (B) Formation of round spheroids in V-bottomed microwells. Still images by time-lapse microscopy. Scale bar, 250 μm. DLD-1 cells were seeded on V-bottomed microwells, which have 1:1, 1.5:1, 2:1, and 3:1 aspect ratios, respectively, and live-imaged for 48 h. (C) The circularity of spheroids that measured every 1 h. Error bars show mean±95%CI. (D) Visualization of nucleus and F-actin of a round spheroid. R2/7 cells expressing WT α-catenin were seeded on V-bottomed microwells, which have a 3:1 aspect ratio, cultured for 48 h, fixed, stained, and imaged. The XZ and YZ view images were produced at the horizontal and vertical lines indicated in the XY view image, respectively, showing 3D round morphology. Scale bar, 20 μm.

In the round-bottomed wells, the projection image of the initial shape from above was circular like the final shape, which was hard to control (Fig. 1 and Fig. 2A). Therefore, developing a new 3D culture model was attempted in which the initial shape could be controlled, and quantitative image analysis is possible. Dispersed cells were seeded into V-bottom rectangular microwells made of nonadhesive polydimethylsiloxane (PDMS) molded in a metal mold (Fig. 2A).

To investigate the relationship between the initial and final shapes of the cell aggregates, spheroid formation experiments with DLD-1 cells were performed using the microwell with several different aspect ratios. The initial shape of the cell mass remained unchanged until about 4–5 h after seeding and changed to a spherical shape by 12–18 h (Fig. 2B and Movie 3). To evaluate this temporal change more objectively, the projected contour of the spheroid in the XY plane was traced, and the temporal change of its circularity was plotted (Fig. 2C). Although the cells were actively oscillating up to 4–5 h after seeding, there was little change in the circularity (the *latent phase* in this report). An increase in circularity was observed between 5 and 18 h, especially up to 12 h (*rising phase*). After that, the circularity remained almost constant (*static phase*). The final circularity and the length of time to reach it were almost constant regardless of the aspect ratio of the initial shape. In this suspension culture system, DLD-1 cells eventually became spherical even if the aspect ratio of the initial shape was large up to 3:1.

Next, the dependence of this morphogenetic process on α-catenin was confirmed (Fig. S3 and Movie 4). As shown in Fig. 2, DLD-1 cell aggregates formed round spheroids (Fig. S3A–C and Movie 4). In contrast, R2/7 cells lacking α-catenin expression showed no change in cell mass shape over time. Stable expression of full-length WT α-catenin (1–906) rescued this abnormality fully (Fig. S3A–C and Movie 4). Although other proteins may be involved in cell adhesion in general, this morphogenesis depends on the cadherin-catenin complex containing α-catenin.

### Detailed analyses of α-catenin tension sensitivity in the process of rounding morphogenesis

**Fig. 3:**
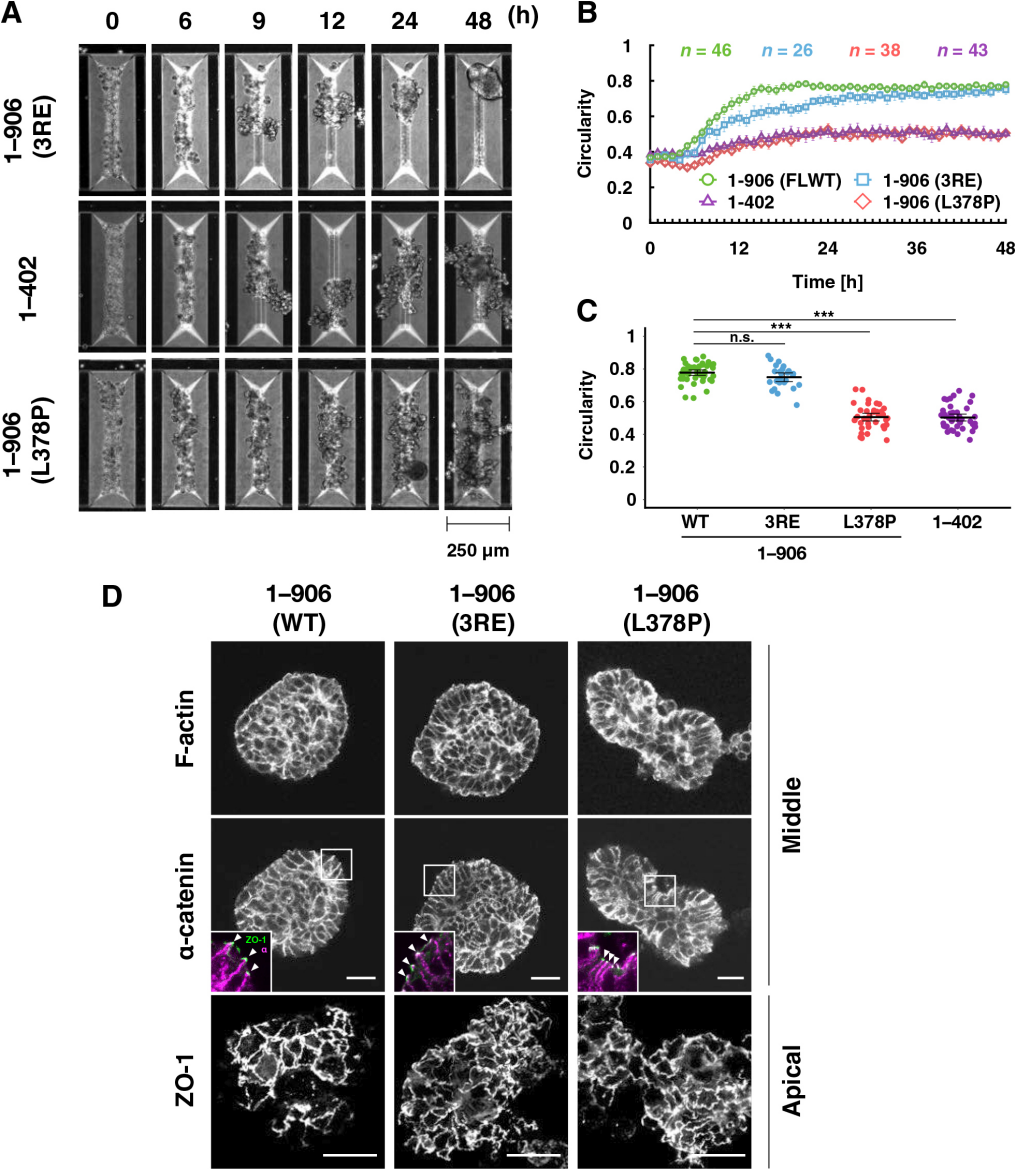
Increased tension sensitivity of α-catenin suppresses increase in circularity of spheroids in V-bottomed microwells. (A) Still images by time-lapse microscopy. Scale bar, 250 μm. R2/7 cells expressing α-catenin mutants with increased sensitivity [1–906 (3RE), top, 1–402, middle, 1–906 (L378P), bottom] were seeded on V-bottomed microwells, respectively, and live-imaged for 48 h. (B) The circularity of the spheroid contour was measured and plotted against time. Error bars show mean±95%CI. (C) The circularity of spheroids at 48 h after seeding. Hypersensitive mutations suppress the increase in circularity. Error bars show mean±95%CI. (***; *P*<0.001, n.s.; not significant.) (D) Visualization of F-actin, FLAG-tagged α-catenin, and ZO-1, showing the proper distribution of F-actin and α-catenin together with tight junction networks revealed with ZO-1. White arrowheads in the inset indicate apical cell-cell junctions. Cells were cultured on V-bottomed microwells for 48 h, fixed, stained, and imaged. ZO-1 images in the bottom panels are maximum intensity projections of 20 slices (20 μm in depth) from the apical surface of spheroids. The display range of pixel intensity was adjusted independently to clarify junctional structures. ZO-1, green; α-catenin, magenta(inset). Scale bars, 20 μm.

First, the effect of mutations that increases the tension sensitivity of α-catenin on the spheroid formation was examined (Fig. 3 and Movie 5). The triple point mutations, 3RE, in which tension sensitivity is increased compared to WT, caused a delay in the shape change, although the final morphology was similar to WT (Fig. 3A and Movie 5). Cells expressing mutant α-catenin with hypersensitivity to tension (L378P or the C-terminal deletion, 1–402) showed insufficient rounding and less smooth contours than WT, respectively. The onset of the rising phase was less clear and the slope of the increase in circularity was smaller than that of the WT (Fig. 3B), and the circularity remained lower than that of the WT for 48 h (Fig. 3C). We visualized cell-cell adhesion in spheroids after 48 h using immunofluorescence staining. TJs were formed in a typical uniform network in WT-expressing cells, indicating that the cortical cell layer of the spheroid is considered a fully developed epithelial sheet (Fig. 3D). Point mutations (3RE and L378P) did not affect the formation of TJs (Fig. 3D). These results quantitatively demonstrate that appropriate tension sensitivity of α-catenin, which allows vinculin release under low tension, is vital for rounding during spheroid formation. The morphogenetic abnormality caused by increased sensitivity is not due to a severe disturbance in the formation of cell-cell adhesion.

**Fig. 4:**
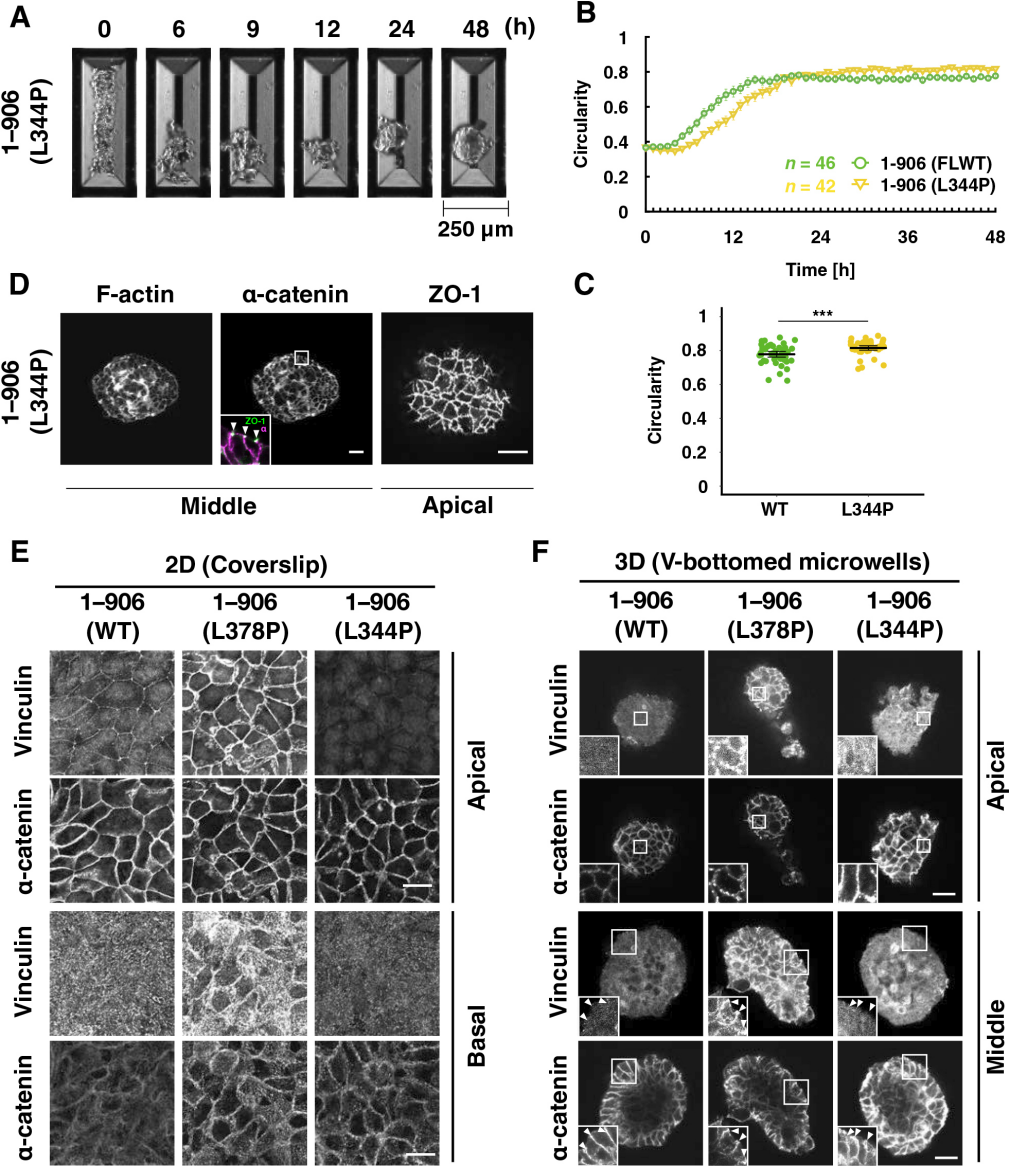
Vinculin does not accumulate at AJs during rounding of WT α-catenin-expressing spheroids on V-bottomed microwells. (A) Still images by time-lapse microscopy. Scale bar, 250 μm. R2/7 cells expressing dull mutant α-catenin [1–906 (L344P)] that lacks vinculin binding ability were seeded on V-bottomed microwells and live-imaged for 48 h. (B) The circularity of the spheroid contour was measured and plotted against time. Error bars show mean±95%CI. (C) The circularity of spheroids at 48 h after seeding. Error bars show mean±95%CI. (***; *P*<0.001.) (D) Visualization of F-actin, FLAG-tagged α-catenin, and ZO-1 showing the proper distribution of F-actin and cadherin together with tight junction networks revealed with ZO-1. White arrowheads in the inset indicate apical cell-cell junctions. ZO-1, green; α-catenin, magenta (inset). Cells were cultured on V-bottomed microwells for 48 h, fixed, stained, and imaged. (E, F) Visualization of Vinculin and FLAG-tagged α-catenin. Cells were cultured on coverslips (E) or V-bottomed microwells (F) for 48 h, fixed, stained, and imaged. In WT spheroids, tension-dependent vinculin accumulation to AJs (white arrowheads) is not obvious, showing less tension applied at AJs. Scale bars, 20 μm.

The vinculin binding-null mutant (L344P; Fig. 4) (Peng *et al.*, 2012) can be interpreted as a dull tension sensitivity mutant that cannot respond to any degree of tension for vinculin binding. This mutation caused a slight delay in the increase in circularity but did form round spheroids (Fig. 4A, B, and Movie 5). The circularity during the static phase was significantly higher in cells expressing α-catenin (L344P) than in WT-expressing cells (Fig. 4C). Cells expressing this mutation also exhibited normal intercellular adhesion formation (Fig. 4D), as did the WT and other mutants (Fig. 3D). These results led us to think that rounding morphogenesis in this system requires very low tension, releasing vinculin from AJs.

In 2D culture, WT-expressing cells showed vinculin accumulation mainly at cell boundaries, especially at vertexes where several cells meet (Fig. 4E). In contrast, there was little vinculin accumulation at cell boundaries in the 3D culture (Fig. 4F). The L378P mutation in α-catenin caused substantial vinculin accumulation at the cell boundary in 2D and 3D cultures, while the L344P mutation did not. Cells cultured in the presence of Blebbistatin from seeding until 48 h showed a similar spheroid formation to untreated cells (Fig. S4 and Movie 7), indicating that the contractile force of myosin II does not contribute much to the spheroid formation in this system. Myosin II activity suppression in cells expressing point mutants (3RE and L378P) caused no obvious difference in morphogenesis compared with untreated cells, indicating that myosin II activity is not involved in morphological abnormalities caused by the mutations. (Fig. S4 and Movie 7).

Alternatively, when actin polymerization and depolymerization were inhibited by cytochalasin D (CytoD) and Jasplakinolide (Jasp), respectively, both inhibitors completely suppressed round spheroid formation (Fig. S5 and Movie 8). It clearly indicates that the round spheroid formation is strongly dependent on actin remodeling.

### The effect of tension sensitivity of α-catenin on the maturation process of AJ

**Fig. 5:**
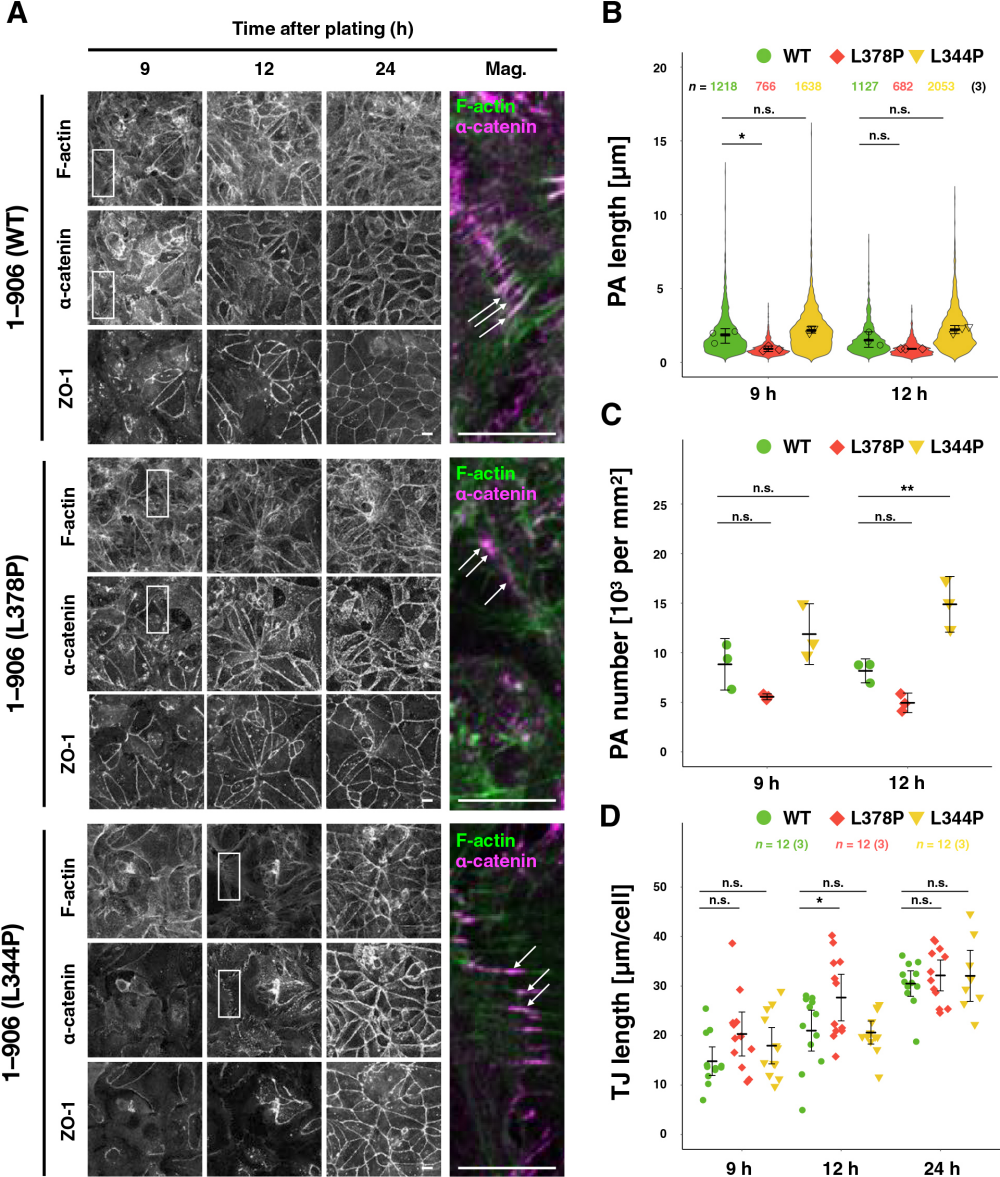
Tension sensitivity mutation of α-catenin alters junctional maturation. (A) Visualization of FLAG-tagged α-catenin (magenta) and ZO-1, and of F-actin (green). R2/7 cells expressing wild-type 1–906 (WT; top), hypersensitive mutant 1–906 (L378P; middle), or dull mutant 1–906 (L344P; bottom) α-catenin were seeded on coverslips, respectively, cultured for 9, 12, 24 h, fixed and stained. White arrows indicate PAs. Scale bars, 20 μm. (B) Measurement of PA length. Violin plots show data distribution, and each dot represents the mean values of each biological replicate. (C) Measurement of PA number normalized by area. Each dot represents the mean values of biological replicates. (D) Measurement of TJ length revealed by ZO-1 normalized by cell number over time, showing fast maturation of TJ network in the hypersensitive mutant (L378P). Each dot represents technical replicates. (B–D) Error bars show mean±95%CI. (*; *P*<0.05, **; *P*<0.01, n.s.; not significant.) See *Materials and Methods* for details of the measurements.

Since a protein molecule α-catenin is an important constituent of the protein complex, AJ, it was hypothesized that abnormal tension sensitivity might affect the process of AJ formation and suppress rounding. To obtain high-resolution images, cell-cell adhesion formation after seeding on coverslips was examined (Fig. 5). As previously observed (Yonemura *et al.*, 1995), cells formed punctate AJs (punctum adherens; PAs) at the early stages after seeding (9–12 h), and then they were reorganized into linear adhesions (zonula adherens; ZAs) (Fig. 5A). The calcium switch experiment was also performed. Broken intercellular adhesions by removal of calcium from the culture medium were reformed by replenishing calcium (Fig. S6). Furthermore, the morphology of PAs stably found at the peripheries of small cell islands was compared (Fig. S7). In total, α-catenin (L344P)-expressing cells formed longer and more PAs, while the length and number of PAs were reduced in α-catenin (L378P)-expressing cells (Fig. 5A–C, Fig. S6A–C, and Fig. S7). The L378P mutation also caused TJs to form earlier (Fig. 5D and Fig. S6D). These results suggest that increased tension sensitivity of α-catenin promotes the maturation of cell-cell adhesion or causes ZA formation, skipping the PA formation process.

### The effect of membrane internalization on epithelial cell morphogenesis

**Fig. 6:**
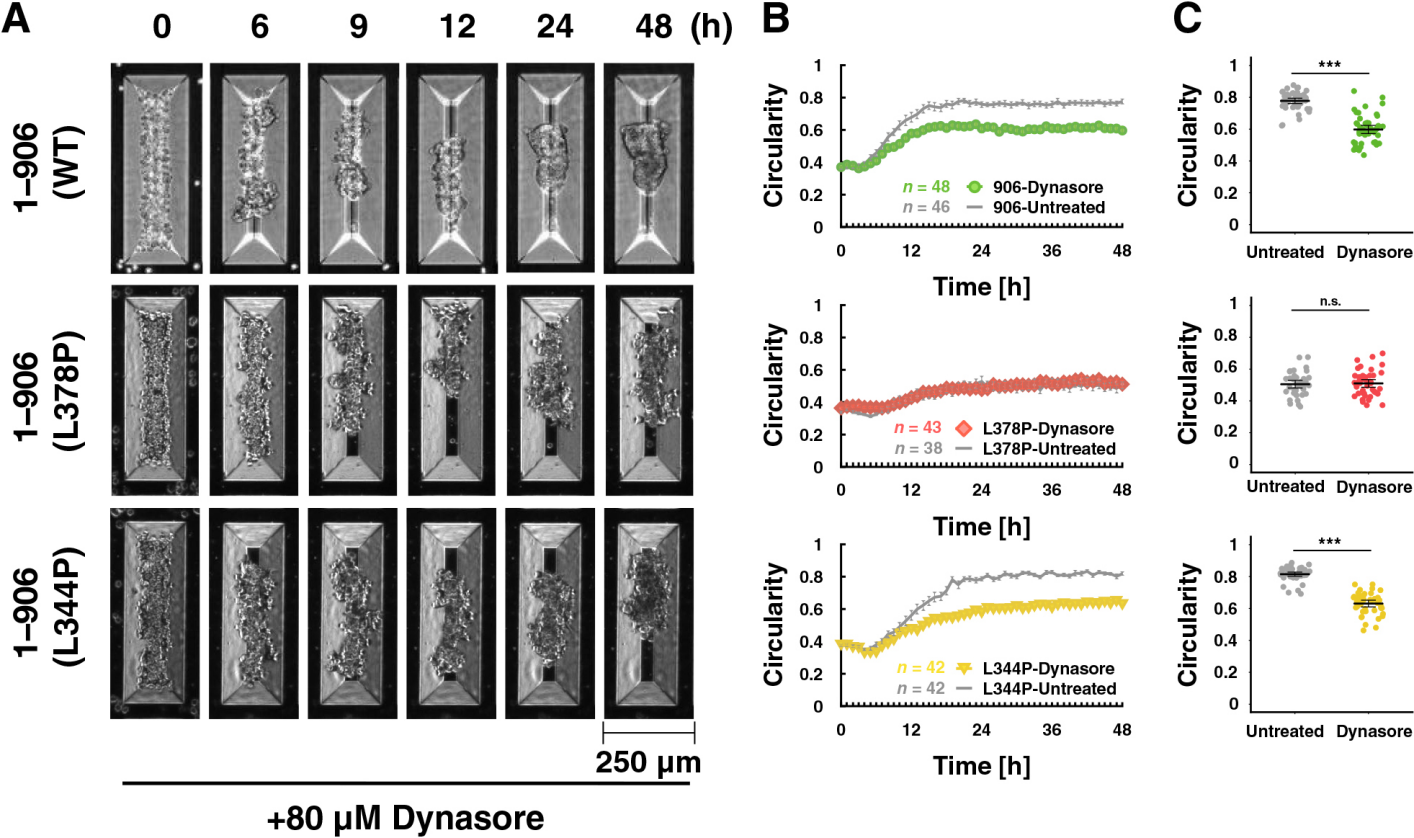
Dynamin inhibition suppresses increase in circularity of spheroids formed in V-bottomed microwells. (A) Still images by time-lapse microscopy. Scale bar, 250 μm. R2/7 cells expressing wild-type (1–906 (WT; top), hypersensitive mutant [1–906 (L378P; middle)], or dull mutant [1–906 (L344P; bottom)] α-catenin were seeded on V-bottomed microwells, respectively, and live-imaged for 48 h in the presence of 80 μM Dynasore, a dynamin inhibitor. (B) The circularity of the spheroid contour was measured and plotted against time. Error bars show mean±95%CI. (C) The circularity of spheroids at 48 h after seeding. Note that in the L378P mutant, a decrease in circularity by Dynasore treatment was not detected. Error bars show mean±95%CI. (***; *P*<0.001, n.s.; not significant.)

Components of cadherin-based cell-cell adhesions, including junctional actin, display a highly dynamic nature at the early stages of adhesion and then are stabilized during junctional development (Huang *et al.*, 2011; Zhang *et al.*, 2005). PAs are reported to be remodeled more dynamically than ZAs (Taguchi *et al.*, 2011). Although there was no clear PA formation in 3D culture (Fig. S8), we speculated that immature AJs were formed during the spheroid formation. Decreased AJ plasticity impairs tissue rearrangements such as cell intercalation (Levayer *et al.*, 2011). Based on these findings, we hypothesized that AJ plasticity is intimately involved in spheroid formation by facilitating multicellular rearrangement. AJ plasticity may be regulated by the internalization of its membrane proteins including E-cadherin, and dynamin GTPase mediates their endocytosis (de Beco *et al.*, 2009; Iyer *et al.*, 2019). Dynamin inhibition by Dynasore caused suppression of rounding for WT α-catenin-expressing cells (Fig. 6A–C – WT and Movie 9), supporting the idea that AJ plasticity is involved in the rounding. In contrast, dynamin treatment did not cause additive suppression on rounding of the L378P mutant-expressing cells (Fig. 6A–C – L378P and Movie 9). Because the C-terminal deletion α-catenin mutant (1–402) bound to vinculin tightly showed very low turnover (Yonemura *et al.*, 2010), it is possible that hypersensitive mutants such as L378P bound to vinculin tightly interfere with internalization of the cadherin-catenin complex through dynamin to the same degree as dynamin inhibition probably due to increased interaction with actin cytoskeleton via vinculin. The L344P mutant lacks vinculin-binding ability. Rounding of this mutant-expressing cells was also inhibited by dynamin (Fig. 6A–C – L344P and Movie 9). These results suggest that the tension sensitivity of α-catenin, especially the vinculin-unbound state of α-catenin is important for AJ plasticity leading to rounding morphogenesis.

### Increased tension sensitivity of α-catenin inhibits collective rather than local cellular rearrangements during round spheroid formation

**Fig. 7:**
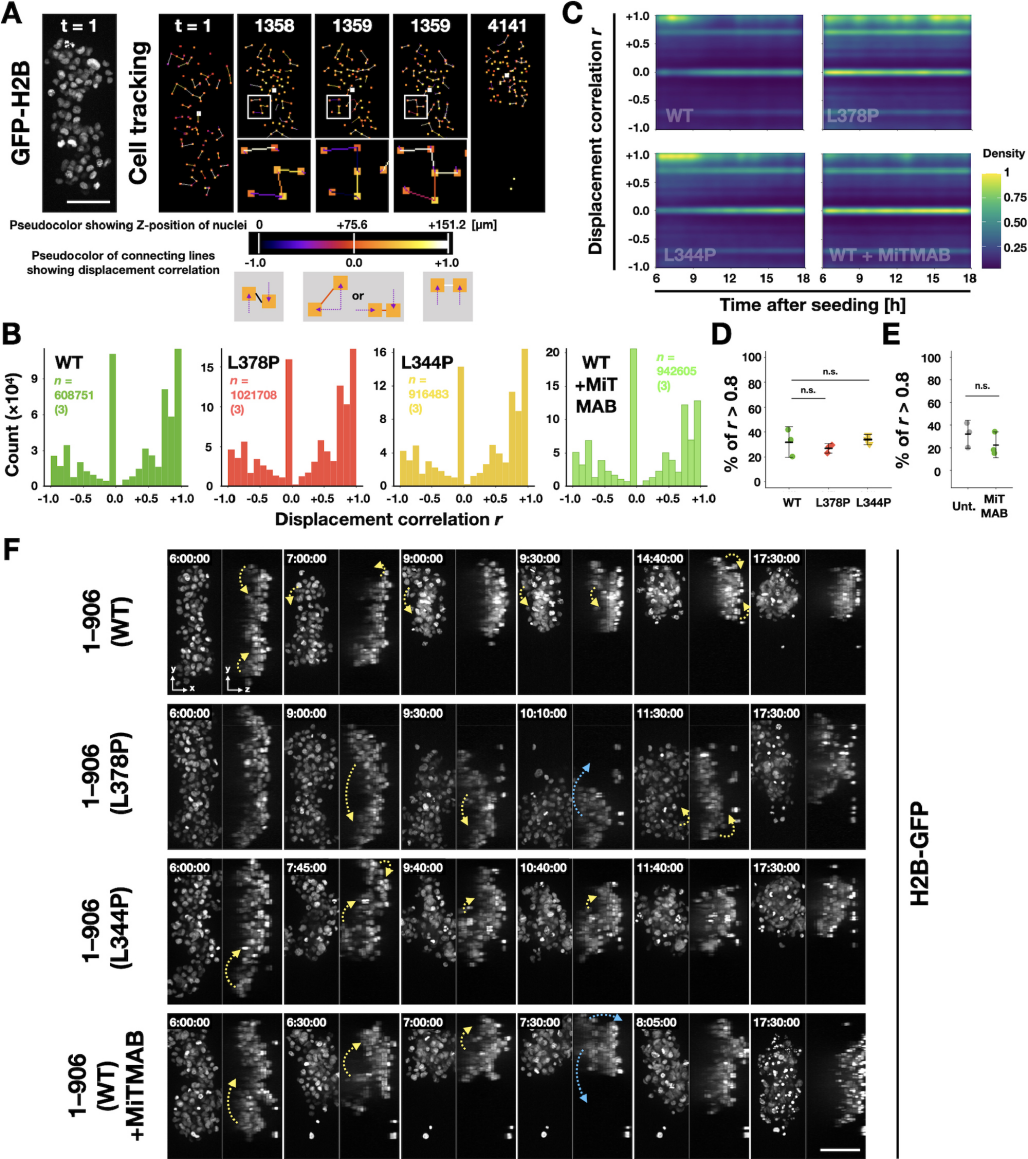
Collective but not single-cell rearrangement is often found in the spheroid rounding. (A) A GFP-H2B image showing nuclei (left) representing the position of cells and visualization of cell tracking analysis process (right). Each cell position in the XY plane is shown in a small square, and its color expresses the depth (‘colder’ color shows a deeper (closer to bottom) position). The displacement of each cell was calculated by comparing its position to that of the previous frame. Then, displacement correlation (*r*) was calculated by comparing 3D displacement vectors of two neighboring cells (connected by lines) and shown by the same color scale (‘colder’ color shows negative, ‘hotter’ color shows a positive correlation, respectively). Frame numbers t are indicated in each image (frame interval=10 s). R2/7 cells expressing H2B-GFP and wild-type [1–906 (WT)] or mutants [1–906 (L378P), or 1–906 (L344P)] α-catenin were seeded on V-bottomed microwells, respectively, cultured for 6 h and then live-imaged for 11.5 h. R2/7 cells expressing H2B-GFP and wild-type α-catenin were also seeded and cultured as well and then live-imaged for the same duration in the presence of 10 μM MiTMAB, a dynamin inhibitor (WT+MiTMAB). (B) Histogram showing data distribution of displacement correlation *r*. (C) Two-dimensional density plot showing data distribution of displacement correlation *r* over time. The density is normalized to 0–1 and coded by pseudo color. (D, E) Percentages of cell displacement pairs *r* greater than 0.8 at between 6–9 hours after seeding. Each dot represents a biological replicate. Error bars show mean±95%CI. (n.s.; not significant.) (F) Still images by confocal time-lapse microscopy. Stack images are shown as maximum intensity projection images of the top (XY) or transverse (YZ) view. The time after seeding is indicated in each frame. Yellow and blue arrows indicate the direction of cell mass movement (‘folding’ and ‘opening,’ respectively). Scale bars, 100 μm.

Then we hypothesized that α-catenin’s tension sensitivity and AJ plasticity are involved in neighbor exchange of cells, like cell intercalation, to drive the rounding of spheroids. Cell nuclei using GFP-tagged Histone 2B were visualized, and cell movements during the rising phase were analyzed (Fig. 7, Movie 10, and Movie 11). To obtain better Z-stack images by confocal microscopy, we utilized amorphous fluoropolymer CYTOP, which has a low refractive index near water, as a substrate of the microwells. By calculating the correlation coefficients (*r*) of the direction of displacement of each cell of a pair of neighboring cells in each frame, we can determine whether the two cells are moving in the same direction with a high positive correlation (0<*r*≤+1) or away from each other with high negative correlation (0>*r*≥–1) (Fig. 7A and Movie 10). The distribution of *r* over the entire period shows two peaks, one near 0 and the other near +1, indicating that the neighboring cells often move in a highly correlated or uncorrelated manner (Fig. 7B). Events in which cells pass by each other with negative *r* do not occur very often. Next, the changes in the distribution of *r* over time were visualized in a 2D density plot with time as the horizontal axis (Fig. 7C). In the two conditions in which rounding proceeded normally (WT and L344P), the proportion of highly correlated movements tended to increase during the first half of the rising phase (6–12 h, especially up to 9 h; Fig. 7C). The distribution of *r* was almost the same in L378P mutant α-catenin-expressing cells that cannot form round spheroids (Fig. 7B). Furthermore, the inhibition of dynamin had almost no effect on these cell movements (Fig. 7B). We note that we used MiTMAB instead of Dynasore to inhibit dynamin because Dynasore was turned out to show phototoxic effects during fluorescent imaging. Under these conditions, there was little change in the distribution over time (Fig. 7C). There was no difference in the percentage of highly correlated movements even when the range was limited to 6–9 h (Fig. 7D, E). These results indicate that this rounding process cannot be explained mainly by local exchanges of neighboring cell positions, detected as movements with negative *r*. Thus, the hypothesis is denied.

To search for other features to focus on, we attempted to simultaneously observe cell movement from the overturned plane using the stacked image data obtained in previous experiments (Fig. 7F, Fig. S9, and Movie 11). As a result, it was observed that in WT α-catenin-expressing cells, long cell aggregates at the initial state repeatedly bent from both ends or at the center and gradually became round (Fig. 7F – WT, Fig. S9A, and Movie 11). Those cell aggregates appear to be constituted of several parts. One part comprises a small number of cells adhering to each other. Those parts move as a group of cells like swing arms, using the region between two parts as a supporting point. When the parts come into contact with each other by bending, they fuse to form a single component. This event is seen as a jump in circularity during the rising phase (Fig. S9). Whenever this fusion happens, AJ remodeling is required; some of the existing AJs should be broken, and new ones should be formed (Fig. S10). The same behavior was observed in cells expressing the L344P mutant (Fig. 7F – L344P and Movie 11). Similar bending motions were observed in cells expressing the L378P mutation and WT cells treated to suppress membrane uptake (Fig. 7F – L378P, Fig. 7F – WT+MiTMAB, and Movie 11). In both cases, cells could not hold the bent state and returned to their original elongated shape after a while (Fig. 7F – L378P, Fig. 7F – WT+MiTMAB, and Movie 11). These results made us imagine that AJ’s low plasticity would suppress such fusion between parts.

**Fig. 8:**
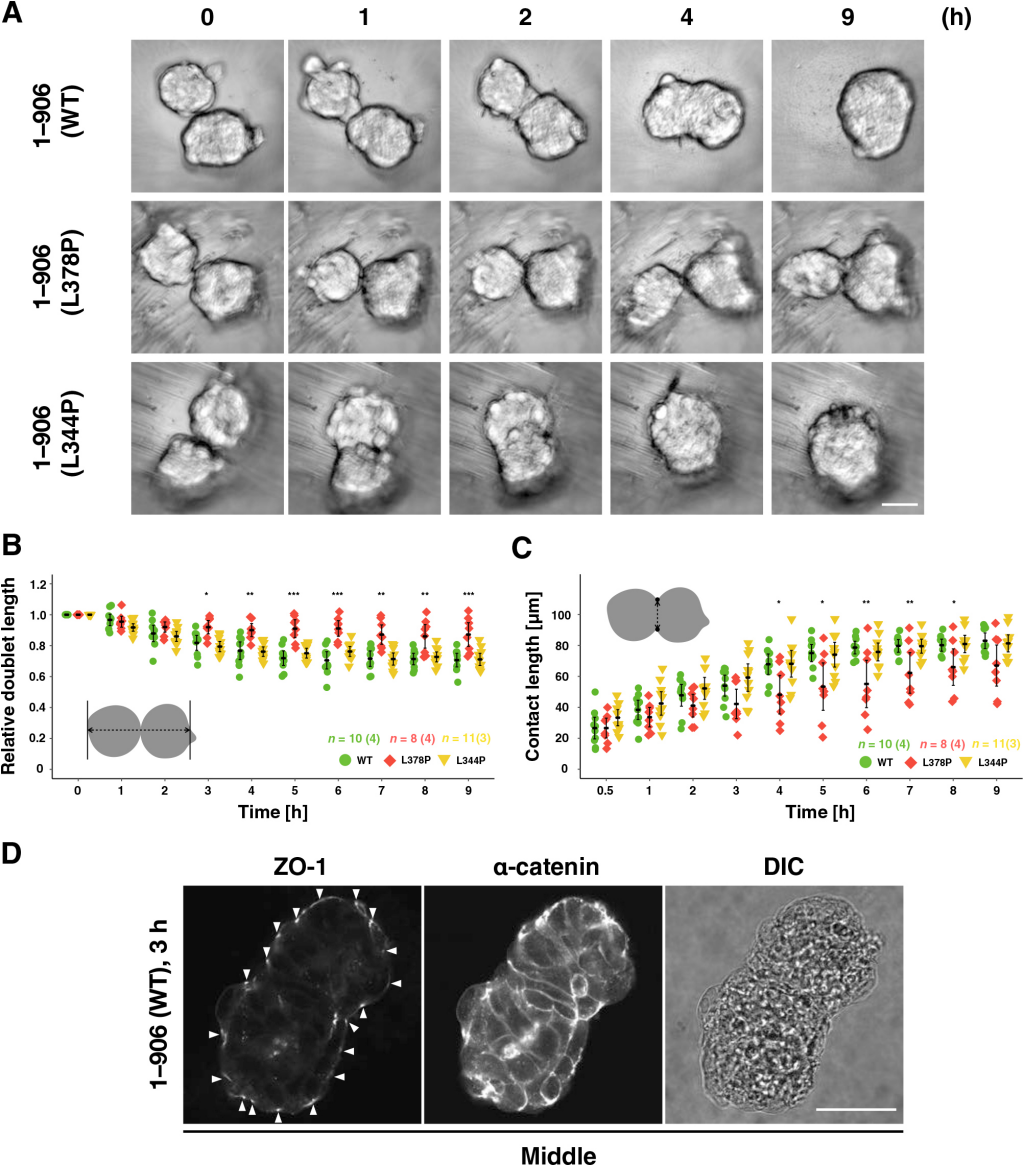
Fusion experiments of spheroids of cells expressing α-catenin with different tension sensitivity. (A) Still images by confocal time-lapse microscopy. A relatively small number (10–20 cells) of R2/7 cells expressing wild-type (1–906 (WT; top), hypersensitive mutant (1–906 (L378P; middle)), or dull mutant (1–906 (L344P; bottom)) α-catenin were seeded on V-bottomed microwells, respectively, cultured for 24 h. Spheroids were then transferred onto V-bottomed wells of 96-well plate and live-imaged for 9 h. Hypersensitive mutation (L378P) suppressed spheroid fusion, suggesting the importance of plasticity of AJ in spheroid fusion. (B) The length of a spheroid pair along the long axis was measured. Any protrusions on spheroids were ignored, and values were normalized to that at 0 h. (C) The length of new contacts between two spheroids was measured as illustrated. (D) Visualization of ZO-1 and α-catenin of a fusing spheroid with a differential interference contrast (DIC) image, showing absence of TJs in the contact area and presence of a surface TJ network. Spheroid pairs were fixed 3 h after contact, mounted in Matrigel, stained, and imaged. (A, D) Scale bars, 50 μm. (B, C) Error bars show mean±95%CI. (*; *P*<0.05, **; *P*<0.01, ***; *P*<0.001, n.s.; not significant.)

**Fig. 9:**
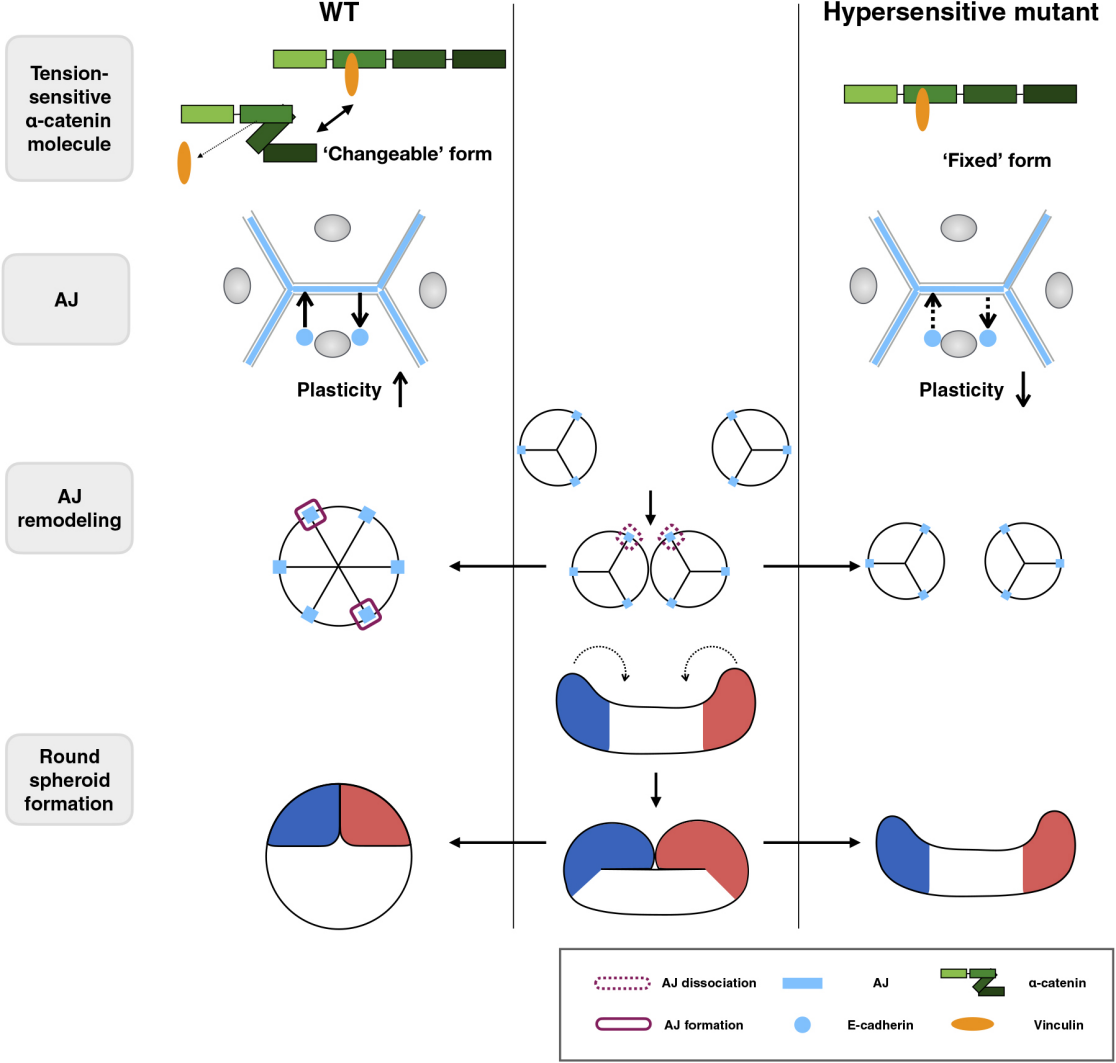
Schematic illustration of the proposed model. Trans-scale relationships from molecular responses to multicellular behaviors during rounding morphogenesis of epithelial spheroid are illustrated. Under suspension conditions, elongated cell aggregates gradually deform into a spherical shape by folding events. It accompanies AJ remodeling (i.e., dissociation and formation), which requires the plasticity of AJs. When α-catenin tension sensitivity is increased to the maximum by mutation, the molecule is fixed to ‘opened’ form, which strongly binds to vinculin. It leads to a decrease in AJ plasticity, prevents AJ remodeling, and results in failure of the collective rearrangement. Thus, proper tension sensitivity of α-catenin ensures rounding morphogenesis of epithelial spheroids.

To test this, we performed fusion experiments of preformed spheroids (Fig. 8, Fig. S10, and Movie 12). In the pair of WT or L344P mutant α-catenin-expressing spheroids, the contact area between them expanded within a few hours after initial contact, forming a single round spheroid (Fig. 8A and Movie 12). In contrast, spheroids expressing the L378P mutation did not develop a clear contact area even after 9 h (Fig. 8A and Movie 12). The L378P mutation suppressed the expansion of the contact area and kept the distance between two spheroids long, whereas the WT and L344P mutants expanded the contact area and shortened the distance between the two spheroids (Fig. 8B, C). Although we could not obtain immunofluorescent images of fusing spheroids with a small contact area due to technical problems, we could observe junctions after the contact area was expanded (3 h after contact). Preexisted TJs at the interface of two spheroids were dissociated and a TJ network was formed at the entire apical surface of fused spheroids, showing that junction remodeling occurs during spheroid fusion (Fig. 8D). Taken together, the appropriate tension sensitivity of α-catenin contributes to the fusion between spheroids, leading to spheroid rounding probably through the AJ remodeling (Fig. 9).

## Discussion

Spheroid formation experiments have been widely performed in biological and medical research (Cavo *et al.*, 2020; Raghavan *et al.*, 2016; Yonemura, 2014). In this study, we established a novel suspension culture model with V-bottomed microwells. Many studies characterized the spheroid formation process using morphology factors such as diameter and circularity (Cavo *et al.*, 2020; Deckers *et al.*, 2018; Kelm *et al.*, 2003; Raghavan *et al.*, 2016; Smeets *et al.*, 2020). The technical advantage of our culture model is that we can precisely define the initial shape with various aspect ratios. Combining the model with image-based quantitative analyses allowed us to define distinct spheroid formation stages and analyze the rounding process at the molecular level.

The minimum requirements for rounding are as follows: 1) the cells adhere to each other with the same strength, 2) they can change their relative spatial positions. A typical example of cellular rearrangements is germ-band extension in the *Drosophila* embryo, where local neighbor exchange of cells is vital (Levayer *et al.*, 2011). In addition, the tissue’s surface tension caused by the cortical tension of individual cells has also been proposed to drive spheroid rounding (Lecuit and Lenne, 2007; Winklbauer, 2015). In this study, physiological tension derived from myosin II was not required for the rounding. Although we do not rule out the possibility of force generation by other myosin family members like myosin-1c (Kannan and Tang, 2018), which is Blebbistatin-resistant, these contributions seem to be low because tension-dependent vinculin recruitment to AJs was not found (Fig. 4). Rather, vinculin release from α-catenin in response to low tension was indispensable. Cell motility necessary for the rounding of spheroids in our system is likely to be based on actin dynamics (Fig. S5).

The suppression of PA formation in the hypersensitive mutant in 2D culture (Fig. 5 and Fig. S6) suggested less AJ plasticity in the mutant because PA is remodeled more dynamic than ZA (Taguchi *et al.*, 2011). Although we assumed first that the low junctional plasticity is shown as decreased neighbor exchanges in cells during spheroid formation, almost no change was detected. This result raises the possibility that cell-cell junctions other than AJs, such as stable desmosomes (Rübsam *et al.*, 2018) suppress cell relocation. Unlike cell-cell intercalation, cells move collectively as gradual folding of a long cell mass, and they fuse to form a round spheroid (Fig. 7 and Movie 11). The hypersensitive mutation unexpectedly interferes with fusion after the folding rather than local cell relocation. The folding should be accompanied by the deformation of a few cells at the hinge region connecting two cell masses. This folding movement can be explained if this is based on actin dynamics consistent with our results. Completing neural tube closure requires the fusion of new contact regions (Nikolopoulou *et al.*, 2017) similarly to our results. The failure of spheroid fusion in the hypersensitive mutant (Fig. 8) further supports the importance of the tension sensitivity of α-catenin for multicellular rearrangement at the cell mass level.

The physiological significance of the tension sensitivity of α-catenin has been reported in the context of the collective migration of epithelial cells. Seddiki *et al.* reported that in MDCK cells, the hypersensitive mutant grown under confinement to patterned areas showed the same cooperativeness of migration as the WT, although the L344P mutant lacking vinculin-binding decreased the cooperativeness (Seddiki *et al.*, 2018). Meanwhile, Matsuzawa *et al.* reported that M319G/R326E double mutations, which increase the tension sensitivity, decrease the cooperativeness of collective cell migration toward a cell-free area in MDCK cells (Matsuzawa *et al.*, 2018). Interpretations of these two reports apparently contradict each other. While Seddiki *et al.* compared distances that cells migrate collectively, Matsuzawa *et al.* focused on the directional coordination of the cell migration to a cell-free area. In the latter case, the cell migration with the direction which is not aligned toward the cell-free area is considered as an ‘uncoordinated’ migration, even if groups of cells migrated collectively. Thus, comparable cell behavior can be interpreted differently by the different criteria. They also contradict our results that cooperativeness of cell movement was almost the same in WT and mutants. One of the important differences is that cells in our 3D culture system do not form obvious focal adhesions found in conventional 2D culture on a stiff substratum. Probably cells move based on amoeboid motility using actin polymerization in our system. Although we tried to eliminate the effects from the cell anchorage to the stiff substratum, focusing on the effects of cell-cell adhesion as already described, the involvement of traction force through cell-substrate adhesion in the tension applied to the cell-cell interface is also important (Maruthamuthu *et al.*, 2011). The degree of its involvement would vary at specific morphogenetic sites of the body depending on the stiffness or constituents of the extracellular matrix. Another difference is that they used established epithelial cell sheets with junctions, whereas, in our 3D culture system, cells are in the process of junction formation. Therefore, a simple comparison of our data and those obtained in a 2D culture system is difficult. Nevertheless, our results highlighted the importance of the tension sensitivity of α-catenin on the AJ remodeling found in spheroid fusion.

Morphogenesis through apical constriction requires strong AJs for force transmission. By contraries, morphogenesis through cell rearrangements requires fast AJ remodeling. Α-Catenin reduces AJ plasticity to ensure the force transmission through binding to vinculin (le Duc *et al.*, 2010; Miyake *et al.*, 2006; Yonemura *et al.*, 2010) under strong tension (>5 pN, corresponding to the force generated by a myosin II molecule) (Maki *et al.*, 2016; Yao *et al.*, 2014) and increases plasticity to allow remodeling through releasing vinculin when the tension is weakened. Considering the results in this study, α-catenin can be regarded as an essential regulator for both types of morphogenesis. Tension sensitivity should be essential for successful switching between these two functions. Furthermore, the tension range for the switching is also important. If α-catenin is bound to vinculin under lower tension, smooth remodeling of AJs would not be possible. Thus, the α-catenin molecule is cleverly designed to respond to the physiological range of tension. Since the direct actin-binding of α-catenin itself is also tension-sensitive, it is necessary to further investigate the relationship between this and the actual adhesion to substrates *in vivo*. For the rapid remodeling of AJs, we consider that the loose association of actin filaments and α-catenin not only at the AJ but also along the lateral membrane is important because the turnover rate of α-catenin at the lateral membrane is much higher than that at AJs, and slowed down severely by a hypersensitive mutation (1–402) (Yonemura *et al.*, 2010). In addition, most of the cadherin-catenin complex including α-catenin distributes along the lateral membrane, serving as a large pool for the AJ remodeling. Hypersensitive mutant α-catenin distributes also mainly along the lateral membrane with tightly bound vinculin, which is likely to decrease the supply of the cadherin-catenin complex from the lateral membrane for AJ formation.

Clonal epithelial cells form round spheroids with high probability *in vitro*, and round embryos are formed from eggs with high precision during development. Tension sensitivity of α-catenin appears to support designed morphogenesis protecting from stochastic fluctuations, which can lead to morphogenetic defects. Another aspect of the importance is the dual roles switched by tension applied to AJs. Binding to vinculin is important for maintaining rigid junctions, which transmit force to cause large deformation of cells. On the other hand, releasing vinculin promotes junctional remodeling while maintaining adhesion among cells. α-Catenin plays a central role in both morphogenetic situations.

## Author contributions

R.N. and S.Y. conceptualization; R.N. and K.K. data curation; R.N. and K.K. formal analysis; R.N. and S.Y. funding acquisition; R.N., M.S., Y.A., and S.Y. investigation; R.N., K.K., M.S., Y.K., and S.Y. methodology; S.Y. project administration; Y.K., M.T., H.M., and Y.Y. resources; K.K. software; S.Y. supervision; R.N. and S.Y. validation; R.N. and K.K. visualization; R.N. writing-original draft; S.Y. writing-review and editing.

## Conflict of Interest

The authors declare that they have no conflicts of interest with the contents of this article.

## Abbreviations

2D: two-dimensional
3D: three-dimensional
AFM: atomic force microscopy
AJ: adherens junction
CE: convergent extension
DAPI: 4',6-diamidino-2-phenylindole
DIC: differential interference contrast
DMEM: Dulbecco’s modified Eagle medium
ECM: extracellular matrix
FBS: fetal bovine serum
HRP: horse radish peroxidase
PA: punctum adherens
PDMS: polydimethylsiloxane
TIRF: total internal reflection fluorescence
TJ: tight junction
ZA: zonula adherens

## Supplementary Material

Movie 1. Effects of tension sensitivity mutation of α-catenin on morphogenesis in round-bottomed wells.

Movie 2. Effects of tension sensitivity mutation of α-catenin on morphogenesis in round-bottomed wells.

Movie 3. Initial aspect ratios of cell mass and final spheroid circularity.

Movie 4. Effects of α-catenin depletion on morphogenesis in V-bottomed microwells.

Movie 5. Effects of tension sensitivity increase of α-catenin on morphogenesis in V-bottomed microwells.

Movie 6. Effects of tension sensitivity decrease of α-catenin on morphogenesis in V-bottomed microwells.

Movie 7. Effects of myosin II inhibition on morphogenesis in V-bottomed microwells.

Movie 8. Effects of actin dynamics inhibition on morphogenesis in V-bottomed microwells.

Movie 9. Effects of Dynamin inhibition on morphogenesis in V-bottomed microwells.

Movie 10. Visualization of cellular tracking during spheroid formation.

Movie 11. Effects of tension sensitivity mutation of α-catenin or Dynamin inhibition on cell behavior during spheroid formation.

Movie 12. Effects of tension sensitivity mutation of α-catenin on spheroid fusion.
