# Supplementary material for: Appropriate tension sensitivity of α-catenin ensures rounding morphogenesis of epithelial spheroids
Source: Cell Struct Funct. 2022 Jun 22;47(2):55–73. doi: 10.1247/csf.22014 (PMC10511042; doi:10.1247/csf.22014)
Supplement: Supplementary file 13 — Supplementary figures [file csf_47_22014_13.pdf]

Supplementary figures

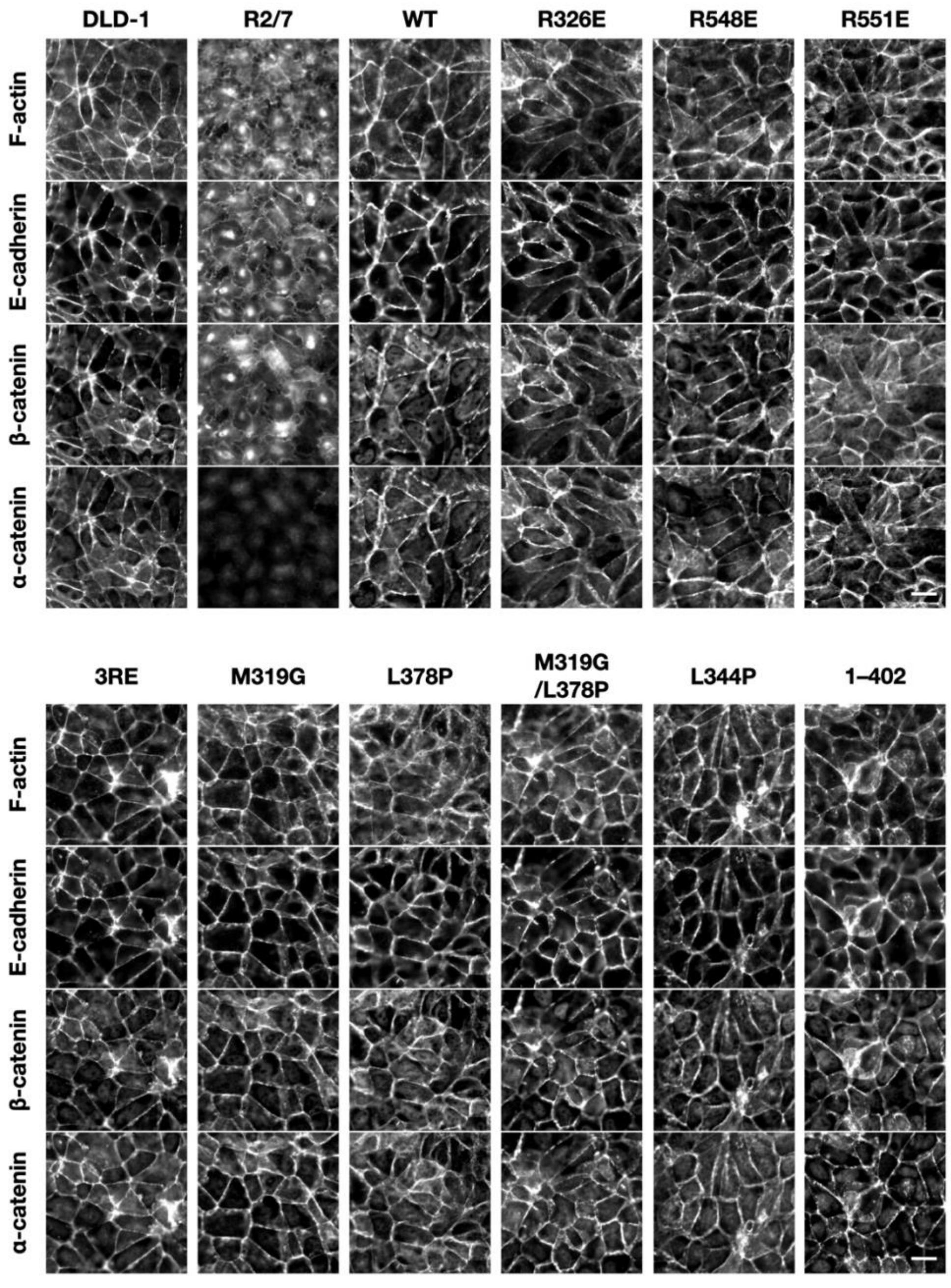

Fig. S1. Cadherin-catenin complex formation and actin filament association to AJs in cell lines used in this study.

(A) Visualization of F-actin, E-cadherin, β-catenin, and α-catenin at the apical junction regions showing the proper distribution of AJ components together with F-actin except for α-catenin-deficient R2/7 cells. α-Catenin was visualized by an anti-α-catenin antibody except for 1–402,

which was visualized by an anti-DDDDK antibody. DLD-1 cells, R2/7 cells, or R2/7 cells expressing WT or mutant α-catenin were seeded on coverslips, cultured for 48 h, fixed, and stained. The display range of pixel intensity was adjusted independently to clarify junctional structures. Scale bars, 20 μm.

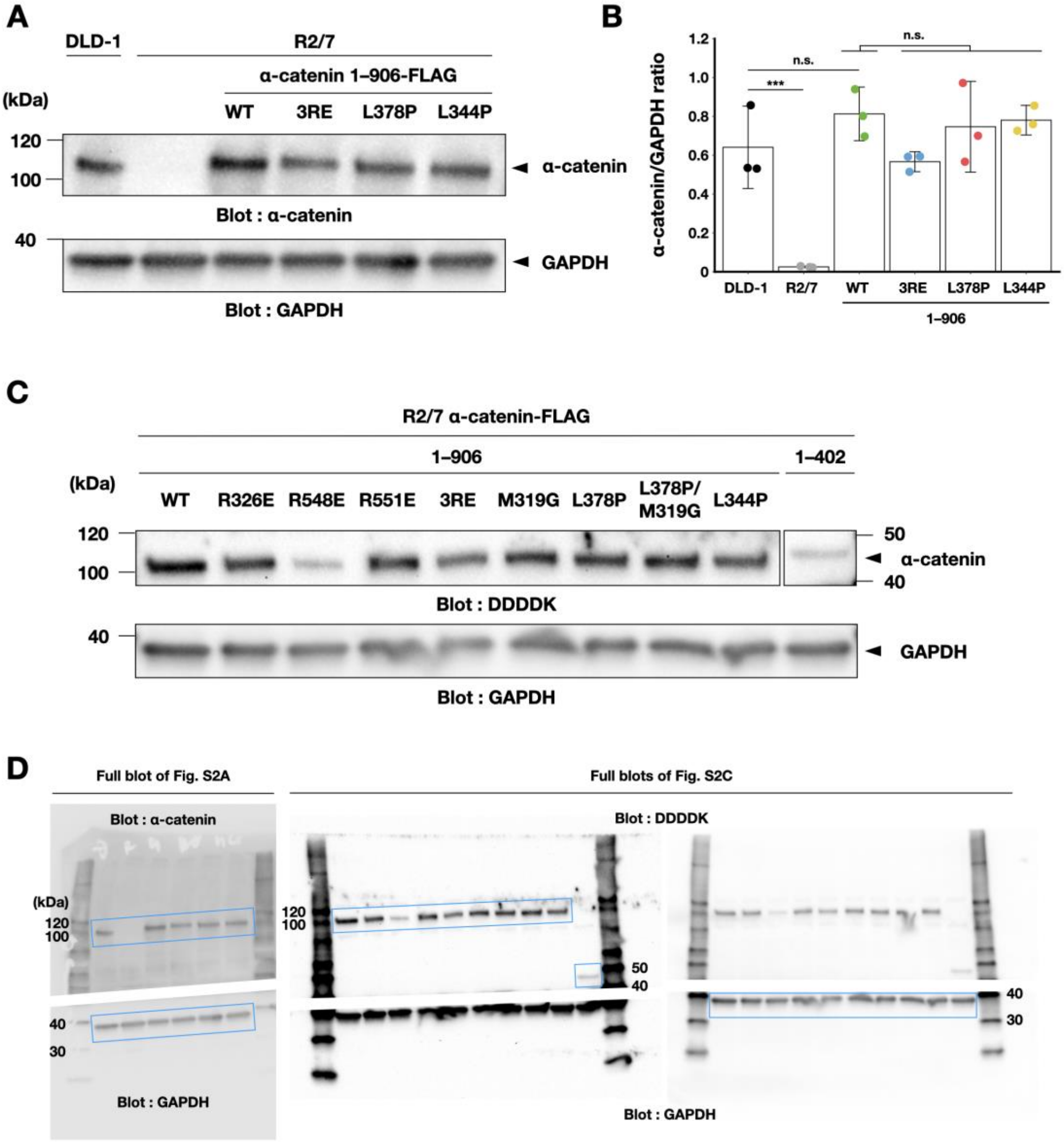

Fig. S2. Expression level of α-catenin in cell lines used in this study.

(A) Blot images by western blotting. DLD-1 cells, R2/7 cells, and R2/7 cells expressing WT or representative mutant α-catenin were cultured for 1 day, and cell lysates were analyzed by immunoblotting with anti-α-catenin and anti-GAPDH antibodies. **(B)** Quantitative analysis of the protein levels of α-catenin in the cell lines. Values are expressed as a ratio to loading control (GAPDH). Each dot represents biological replicates. Error bars show mean ± 95%CI. (***; *P*<0.001.) (**C**) Blot images by western blotting. R2/7 cells expressing WT or mutant α-catenin were cultured as well, and the lysates were analyzed by immunoblotting with anti-DDDDK tag and anti-

GAPDH antibodies. (**D**) Uncropped images of **A** and **C**. Cropped regions are indicated by blue lines. Membranes were cut in half horizontally, and each piece was separately detected by indicated antibodies. Note that the batch of cells used here was different from other cell biological experiments.

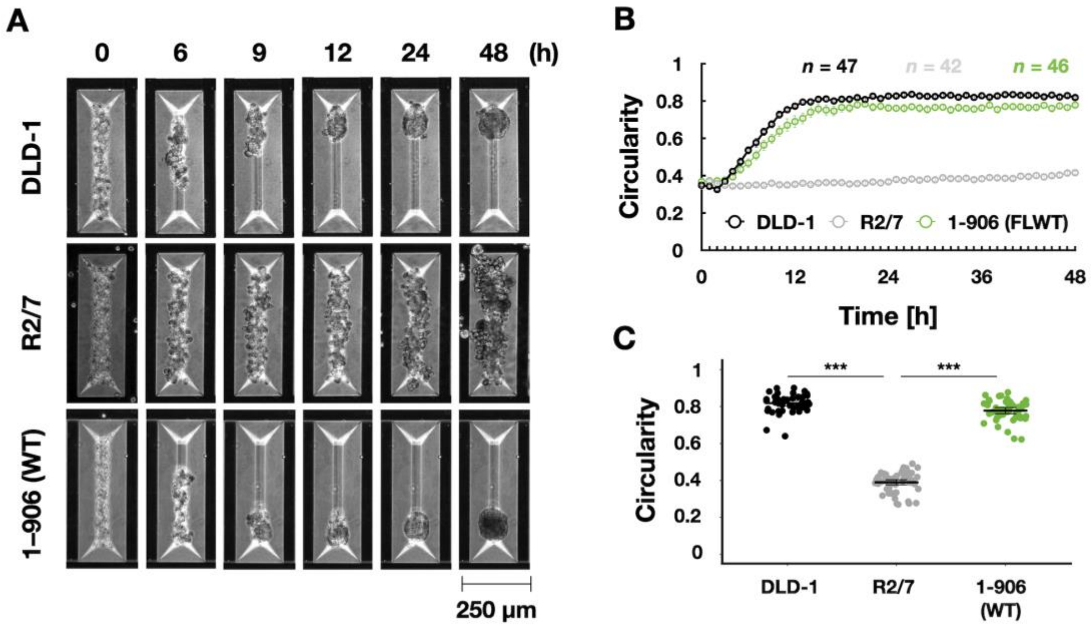

Fig. S3. Spheroid formation in V-bottomed microwells and its quantitative analysis.

(A) Still images by time-lapse microscopy. DLD-1 cells (top), R2/7 cells (middle), or R2/7 cells expressing WT α-catenin (1–906; bottom) were seeded on V-bottomed microwells, respectively, and live-imaged for 48 h. Cells expressing WT α-catenin form round spheroids even from an initial rectangular shape with a high aspect ratio. Scale bar, 250 μm. **(B)** The circularity of the spheroid contour was measured and plotted against time. The cadherin-catenin complex function based on α-catenin expression is essential for an increase in circularity. Error bars show mean ± 95%CI. **(C)** The circularity of spheroids at 48 h after seeding. Error bars show mean ± 95%CI. (***; $P<0.001$.)

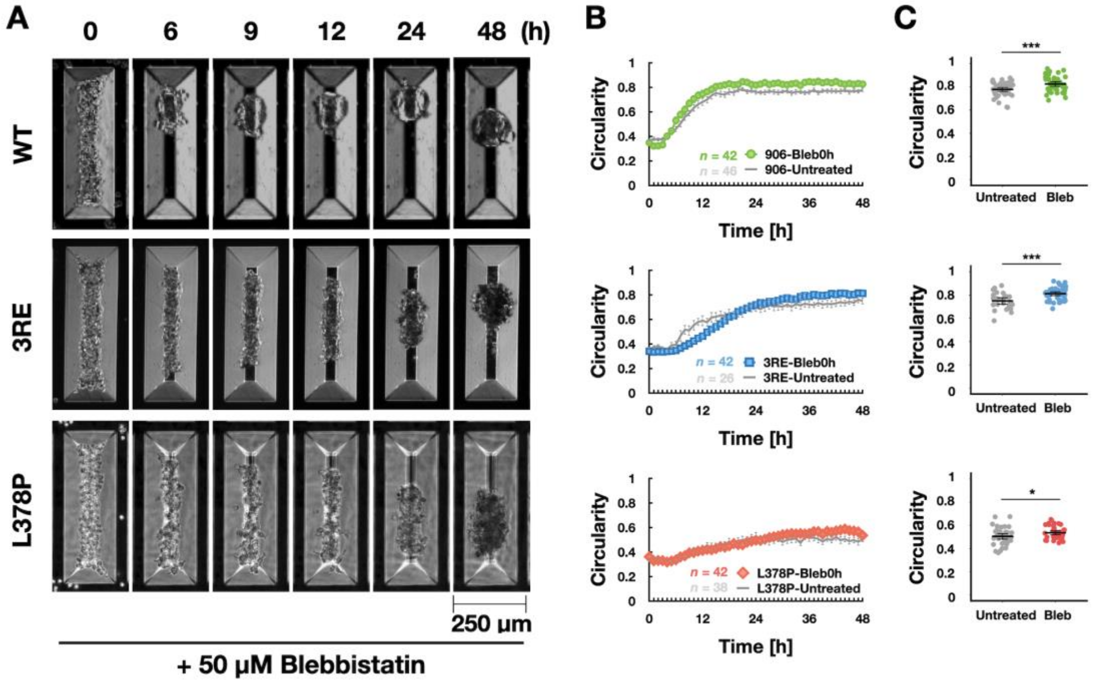

Fig. S4. Myosin II inhibition does not alter circularity changes of spheroids of both WT and mutant cells.

(A) Still images by time-lapse microscopy. R2/7 cells expressing wild-type (1-906 (WT; top), hypersensitive mutants [1–906 (L378P; middle) or 1–906 (3RE; bottom)] α-catenin were seeded on V-bottomed microwells, respectively, and live-imaged for 48 h in the presence of 50 µM Blebbistatin. Scale bar, 250 µm. **(B)** The circularity of spheroids that measured every 1 h. Error bars show mean ± 95%CI. **(C)** The circularity of spheroids at 48 h after seeding. Error bars show mean ± 95%CI. (*; *P*<0.05, ***; *P*<0.001.)

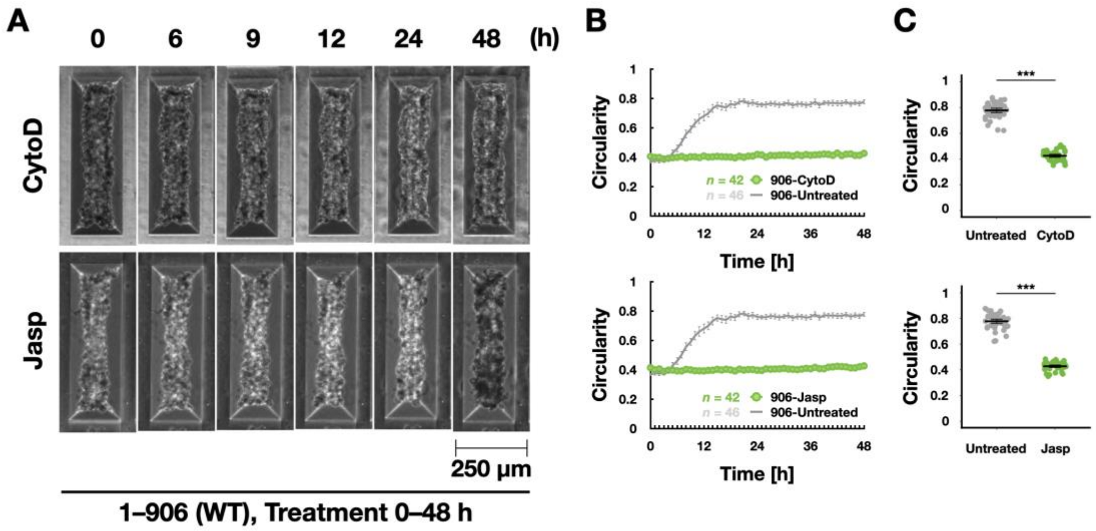

Fig. S5. Inhibition of actin remodeling completely suppresses round spheroid formation.

(A) Still images by time-lapse microscopy. Scale bar, 250 µm. R2/7 cells expressing WT α-catenin were seeded on V-bottomed microwells and live-imaged for 48 h in the presence of 20 µM actin polymerization inhibitor, Cytochalasin D (CytoD; top) or 2 µM actin filament stabilizer, Jasplakinolide (Jasp; bottom), respectively. **(B)** The circularity of spheroids that measured every 1 h. Error bars show mean ± 95%CI. **(C)** The circularity of spheroids at 48 h after seeding. Error bars show mean ± 95%CI. (***; P<0.001.)

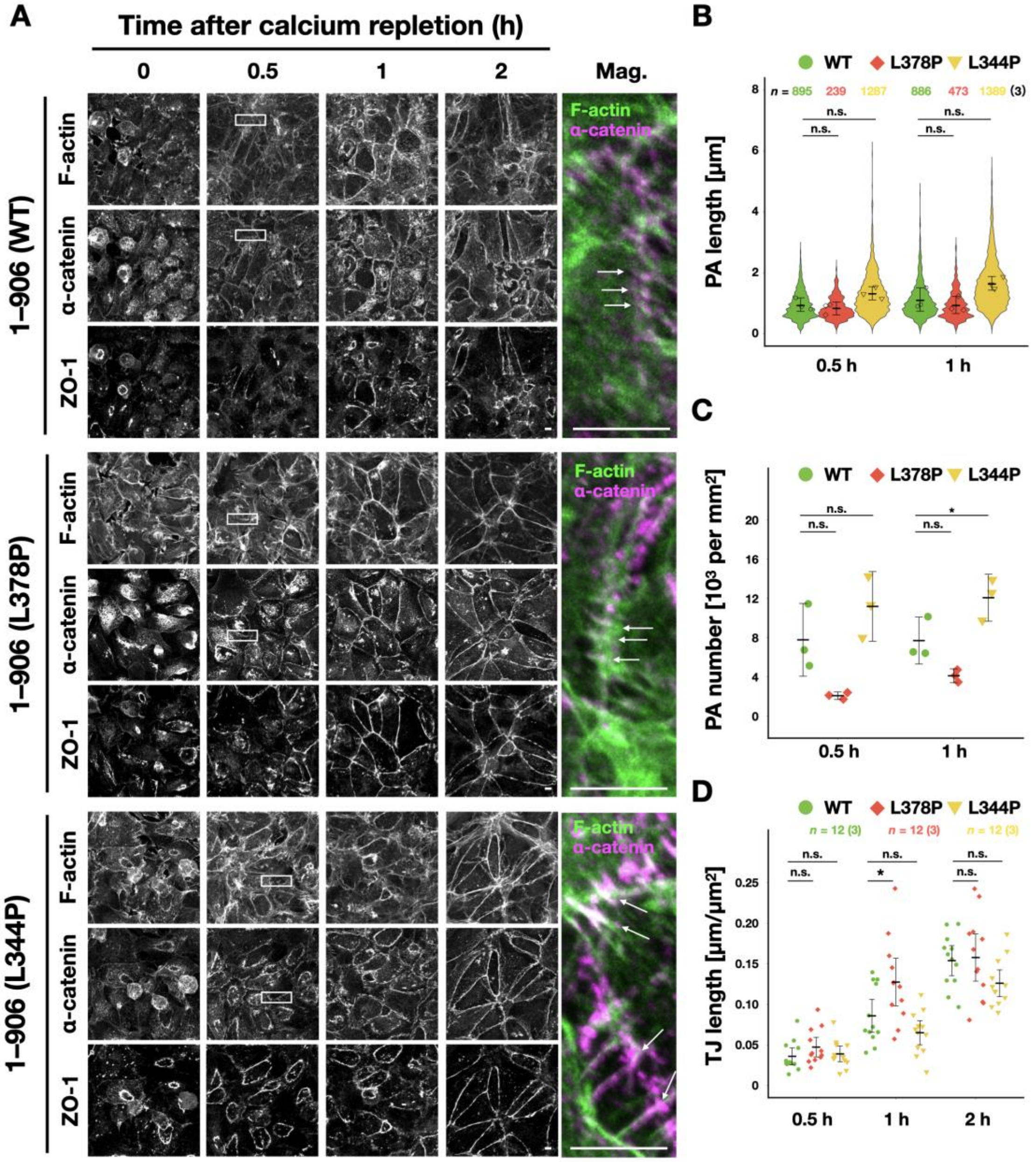

Fig. S6. Tension sensitivity mutation of α-catenin affects the process of junctional formation after calcium switch.

(A) Visualization of F-actin, FLAG-tagged α-catenin, and ZO-1. R2/7 cells expressing wild-type [1–906 (WT; top)], hypersensitive mutant [1–906 (L378P; middle)], or dull mutant [1–906 (L344P; bottom)] α-catenin were seeded on coverslips, respectively, cultured for 24 h in normal medium, and for another 4 h in low calcium medium to dissipate cell-cell adhesion. Then, cells were again cultured in a normal medium for 0.5, 1, 2 h, fixed, and stained to compare the cell-cell adhesion

formation process. White arrows indicate PAs. (**B**) Measurement of PA length. Violin plots show data distribution, and each dot represents the mean values of each biological replicate. **(C)** PA number normalized by area. Each dot represents biological replicates. **(D)** Measurement of TJ length normalized by cell number over time**.** Each dot represents biological replicates. Scale bars, 5 μm. (**B–D**) Error bars show mean ± 95%CI. (*; *P*<0.05, n.s.; not significant.) See *Materials and Methods* for details of the measurements.

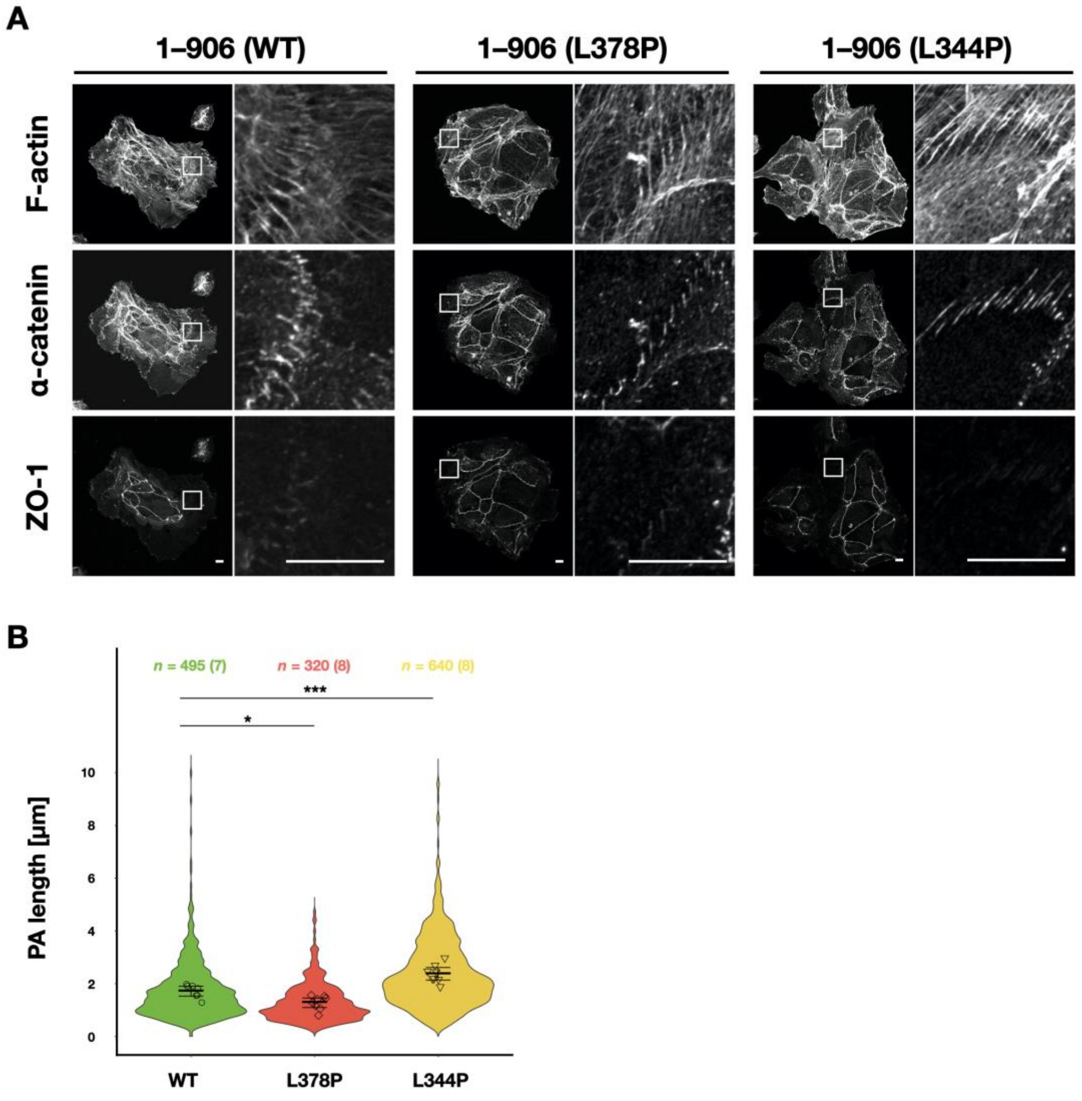

Fig. S7. Tension sensitivity mutation of α-catenin alters junctional formation at cell island peripheries.

(A) Visualization of F-actin, FLAG-tagged α-catenin, and ZO-1. R2/7 cells expressing wild-type (1–906 (WT; left), hypersensitive mutant [1–906 (L378P; middle)], or dull mutant [1–906 (L344P; right)] were seeded on coverslips, respectively, cultured for 24 h, fixed, and stained. Scale bars, 10 µm. **(B)** Measurement of PA length. Violin plots show data distribution, and each dot represents the mean values of each biological replicate. Error bars show mean ± 95%CI. (*; *P*<0.05, ***; *P*<0.001.) See *Materials and Methods* for details of the measurement.

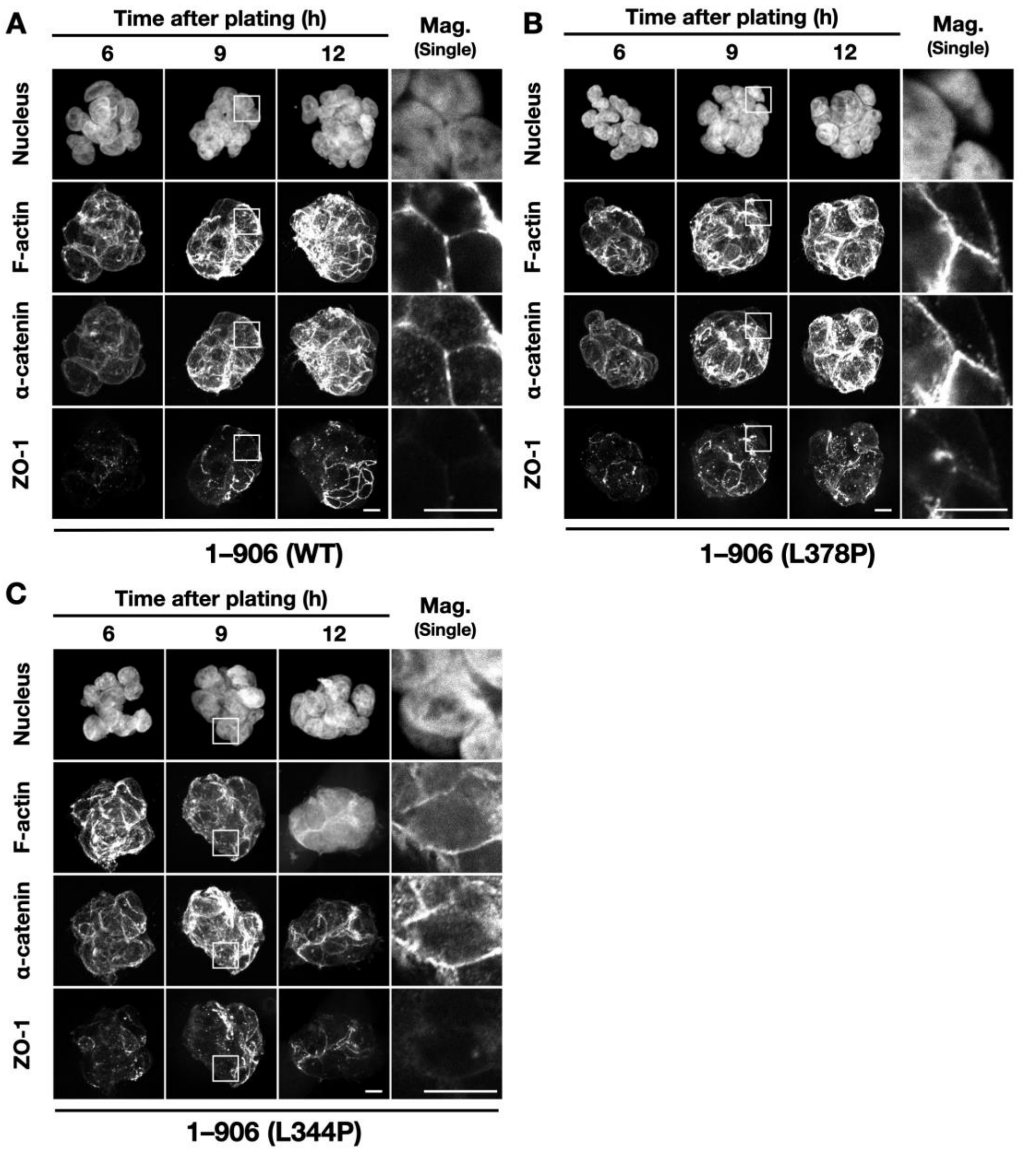

Fig. S8. Effects of tension sensitivity mutation of α-catenin on the junctional formation of spheroids formed on Matrigel.

(A–C) Visualization of the nucleus, F-actin, FLAG-tagged α-catenin, and ZO-1 showing cell-cell junction formation process under 3D culture condition. Images are shown as maximum intensity projections of the entire Z-stack except for magnified images (Mag.), which are single slices of the region of the white box. R2/7 cells expressing wild-type [1–906 (WT; **A**)], hypersensitive mutant [1–906 (L378P; **B**)], or dull mutant [1–906 (L344P; **C**)] were seeded on Matrigel, respectively,

cultured for 6, 9, 12 h, fixed, and stained. Scale bars, 10 μm.

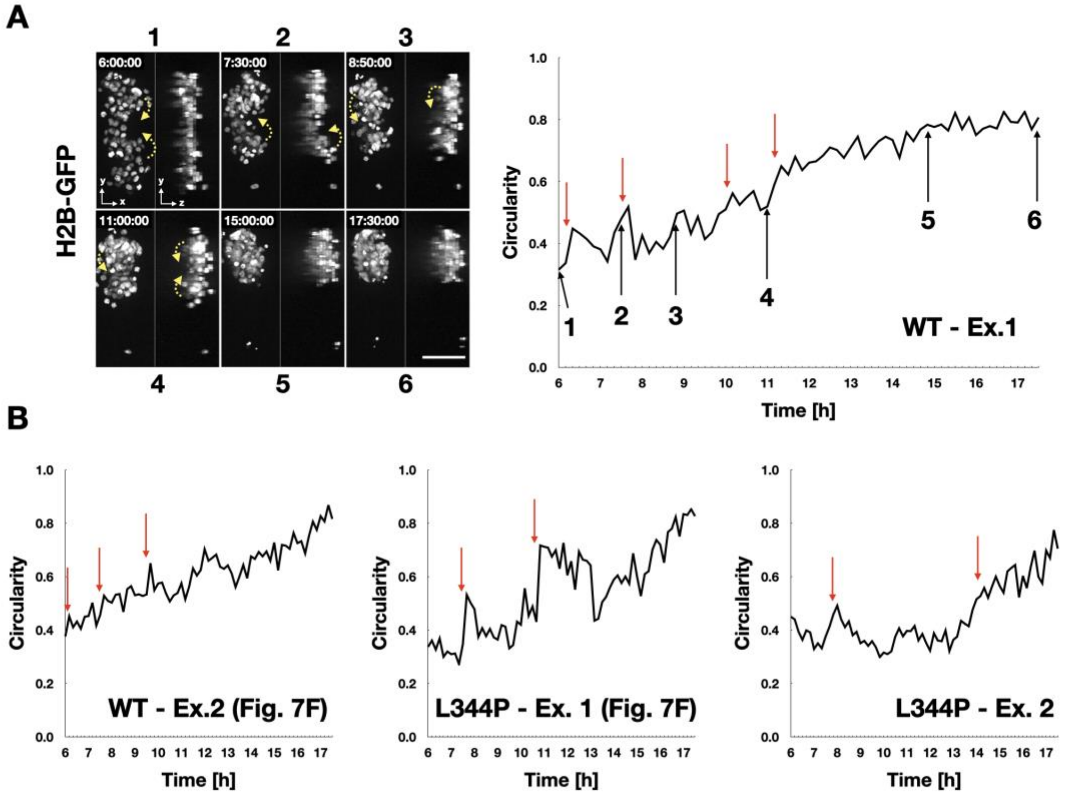

Fig. S9. Contribution of folding movement of cell mass to circularity increase.

(A) Still images by confocal time-lapse microscopy (left) and the circularity of spheroids that measured every 10 min (right). Stack images are shown as maximum intensity projection images of the top (XY) or transverse (YZ) view. The time after seeding is indicated in each frame. Yellow arrows indicate the direction of cell mass movement. Scale bar, 100 µm. Each time point indicated in the image is also shown in the graph (black arrows). Red arrows show timings of a large increase in circularity. R2/7 cells expressing H2B-GFP and WT α-catenin were seeded on V-bottomed microwells, respectively, cultured for 6 h, and then live-imaged for 11.5 h. The folding movement was often observed in the early time (6–12 h) of the rising phase, not in later time (12–17.5 h), consistent with the timing of the increase in high correlation movement in **Fig. 7C**. (**B**) Other examples of the circularity changes of spheroids that measured every 10min.

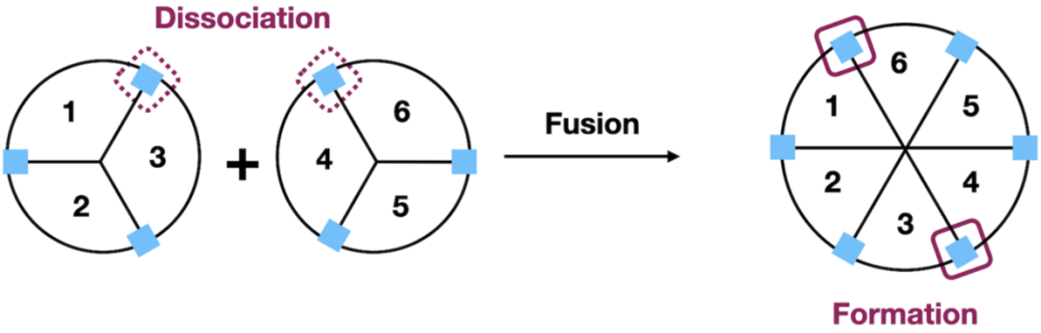

Fig. S10. Schematic drawing of cellular rearrangement during fusion of two multicellular parts.

The necessity of junctional remodeling (i.e., dissociation and formation) for the fusion of two spheroids is shown. Cell-cell junctions (blue square) between cells numbered 1–3 and 4–6 are not found in the fused resultant spheroid and should have been dissociated, and then new junctions should be formed between cells 1–6 and 3–4, respectively.
